# 3D-patterned inverse-designed mid-infrared metaoptics

**DOI:** 10.1038/s41467-023-38258-2

**Published:** 2023-05-13

**Authors:** Gregory Roberts, Conner Ballew, Tianzhe Zheng, Juan C. Garcia, Sarah Camayd-Muñoz, Philip W. C. Hon, Andrei Faraon

**Affiliations:** 1 Kavli Nanoscience Institute and Thomas J. Watson Sr. Laboratory of Applied Physics, California Institute of Technology, 1200 E California Blvd, Pasadena, 91125 CA USA; 2 NG Next, Northrop Grumman Corporation, 1 Space Park Drive, Redondo Beach, 90278 CA USA; 3 Present Address: Jet Propulsion Laboratory, California Institute of Technology, 4800 Oak Grove Dr, Pasadena, 91109 CA USA; 4 Present Address: Applied Physics Laboratory, The Johns Hopkins University, 11100 Johns Hopkins Road, Laurel, 20723 MD USA

**Keywords:** Sub-wavelength optics, Nanophotonics and plasmonics, Imaging and sensing, Mid-infrared photonics, Metamaterials

## Abstract

Modern imaging systems can be enhanced in efficiency, compactness, and application through the introduction of multilayer nanopatterned structures for manipulation of light based on its fundamental properties. High transmission multispectral imaging is elusive due to the commonplace use of filter arrays which discard most of the incident light. Further, given the challenges of miniaturizing optical systems, most cameras do not leverage the wealth of information in polarization and spatial degrees of freedom. Optical metamaterials can respond to these electromagnetic properties but have been explored primarily in single-layer geometries, limiting their performance and multifunctional capacity. Here we use advanced two-photon lithography to realize multilayer scattering structures that achieve highly nontrivial optical transformations intended to process light just before it reaches a focal plane array. Computationally optimized multispectral and polarimetric sorting devices are fabricated with submicron feature sizes and experimentally validated in the mid-infrared. A final structure shown in simulation redirects light based on its angular momentum. These devices demonstrate that with precise 3-dimensional nanopatterning, one can directly modify the scattering properties of a sensor array to create advanced imaging systems.

## Abstract (web summary)

The authors show computationally optimized, multilayer scattering structures in the mid-infrared for high efficiency imaging. Multispectral and polarization sorting devices are fabricated via two-photon lithography and characterized optically.

## Introduction

Nanophotonics synthesizes the study of light-matter interaction with the precise, repeatable techniques of nanofabrication. For example, dielectric metasurfaces are arrays of subwavelength scatterers that apply a spatially varying phase, polarization or amplitude response to an incoming wavefront^1^. The local control is related to the specific shape of each scatterer which can be chosen to compactly replicate and combine functionalities of common optical components like lenses, beamsplitters, polarizers, and waveplates or realize more novel devices such as those used for visible color routing at the pixel level^2^. For metasurfaces, the absence of substantial inter-element electromagnetic coupling is often leveraged for ease of design, but this simplification also limits the available degrees of freedom. Ultimately, we would like to tailor unique scattering behaviors for wavefronts with different spectral, spatial, and polarization properties. To do this, we can expand the design space to volumetric devices where a material is patterned at subwavelength resolution in three dimensions.

Three-dimensional (3D) devices take advantage of a larger set of optical modes to achieve unprecedented performance in a variety of tasks, but require an efficient gradient-based optimization algorithm based on full-wave electromagnetic simulation. Searching the high-dimensional space of permittivity profiles, typically for a local optimum to an electromagnetic merit function, is referred to as inverse design^3–6^. In this area, quasi-2D on-chip photonic devices have been explored extensively where patterning in the direction of light propagation is achieved in a single fabrication layer^7–9^. The fully 3D design paradigm for free space applications is yet to emerge in the infrared and visible spectra, mostly due to the increased fabrication complexity of volumetric devices. However, early works in this area utilizing one- and two-layer processes for optical applications or many-layer microwave prototypes have shown the utility of moving to thicker devices^10–12^. In this work, we optimized a two-photon polymerization (TPP) lithography process to create multilayer structures at optical wavelengths. This technique has been employed in the past for fabricating refractive, diffractive, gradient index, and extruded 2D inverse-designed optical components^13–16^. By exploiting TPP flexibility for 3D patterning at subwavelength resolution, we experimentally demonstrated multiple inverse-designed, multilayer photonic devices with applications to advanced imaging in the mid-infrared band (3–6 μm).

**Fig. 1:**
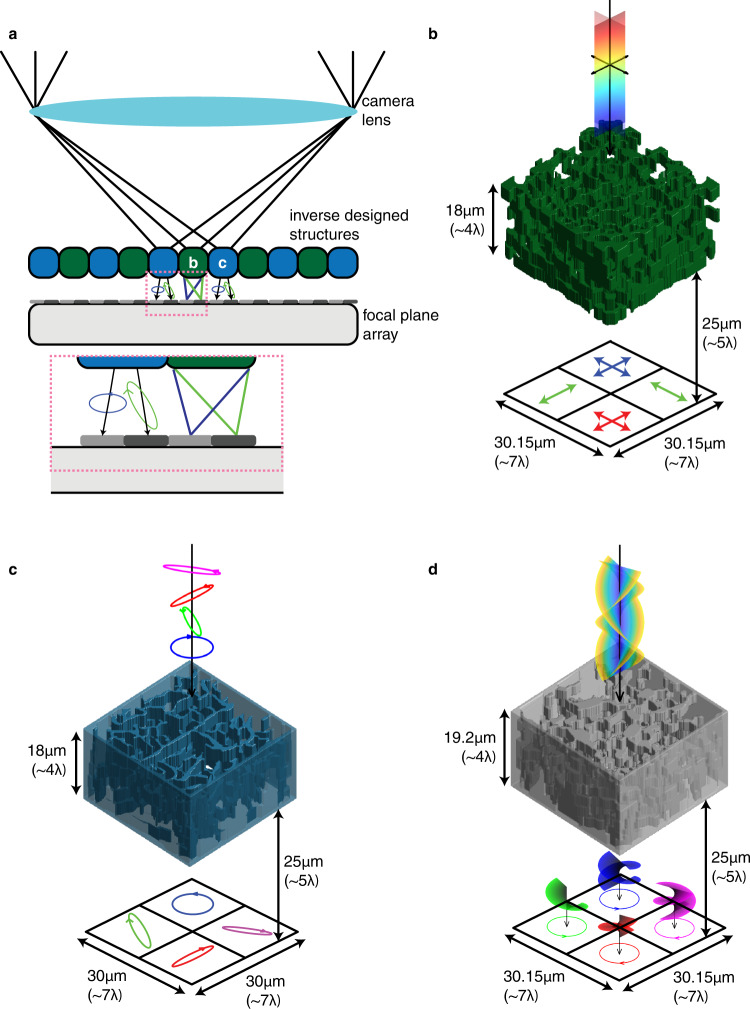
Conceptual depiction of devices in this work. **a** 2D cross section schematic of camera with inverse designed scattering elements placed on top of photosensitive elements at the focal plane of the imaging lens. Green elements sort by color and blue elements sort by polarization, shown in more detail in (**b**, **c**). **b** Rendering of multispectral and linear polarization device that sorts three bands of wavelengths with the middle band further split on polarization. **c** Rendering of full Stokes polarimetry device that sorts four analyzer Jones vectors to different quadrants. **d** Rendering of angular momentum splitting device that sorts combinations of orbital angular momentum (*l*) and spin (*s*) degrees of freedom.

Compact imaging systems utilize wavelength- and polarization-selective elements to characterize fundamental properties of wavefronts. Color imaging in consumer cameras follows this approach where absorptive filters are placed on top of collections of pixels to sense three or four spectral overlaps. The classic arrangement, referred to as a Bayer pattern, consists of a red, blue, and two green filters arranged 2 × 2 in a square^17^. Filtering schemes like this come at a cost of transmission efficiency because they absorb all light outside of their passband leading to average transmission values of ~33% under uniform spectral illumination. Solutions to this problem have converged on the concept of color routing where scattering structures accept light incident on a group of pixels and redirect each wavelength band to a different pixel^2,12,18–20^. In this manuscript, we demonstrate an efficient, multilayer inverse-designed device in the mid-infrared for accomplishing this task and further augment it to sense linear polarization. Beyond multispectral imaging, the geometry of splitting light at the focal plane, depicted in Fig. 1a, can be tailored to efficiently decode other electromagnetic properties. Designing at the pixel level modularizes the optical system, allowing focal plane arrays equipped with arrangements of scattering structures to control the imaging properties of the camera. Figure 1b–d indicates the breadth of devices in this manuscript.

## Results

### Multispectral and polarization sorting

The first application we explored is combined multispectral and polarization imaging. Absorption spectra in the mid-infrared, part of the molecular fingerprinting region^21^, correlate strongly to distinct chemical species. Among many areas of interest, this can be used for environmental monitoring^22,23^ and biomedical imaging^24,25^. Solutions such as multiplexed filters in the mid-infrared suffer from low overall transmission efficiency^26^. They also lack a straightforward path towards multifunctionality that may be critical for a given application. In remote thermal monitoring, for example, multispectral and polarization filtering can be used in tandem to distinguish radiated and reflected light reducing instances of thermal blindness^27^. To address these challenges, we designed and fabricated a multilayer color-routing device with additional linear polarization discrimination.

The optimization goal, stated in Eq. (1), is constructed to sort three spectral bands from 3.7–5 μm and distinguish between linear polarization for the middle band. The device dimensions are 30.15 μm × 30.15 μm × 18 μm (6.6 × 6.6 × 4.0 ${\lambda }_{{{{{{{{\rm{mid}}}}}}}}}^{3}$), broken into six 3 μm thick layers, compact enough to be tiled on a high-resolution focal plane array.

1 $$\begin{array}{r}\mathop{\max }\limits_{{{{{{{{\boldsymbol{\epsilon }}}}}}}}\in {\{{\epsilon }_{\min },{\epsilon }_{\max }\}}^{N}}g({{{{{{{\bf{E}}}}}}}})=\mathop{\sum}\limits_{\lambda }S\left(\left(\mathop{\sum}\limits_{p}\mathop{\sum}\limits_{q}\kappa (q,p,\lambda )\frac{{I}_{p}({{{{{{{{\bf{r}}}}}}}}}_{q},\lambda )}{{I}_{\max }(\lambda )}\right);k\right)\\ {I}_{p}({{{{{{{{\bf{r}}}}}}}}}_{q},\lambda )=||{{{{{{{{\bf{E}}}}}}}}}_{p}({{{{{{{{\bf{r}}}}}}}}}_{q},\lambda )|{|}^{2}\end{array}$$

Electromagnetic inverse design that utilizes the mathematical adjoint method for calculating gradients with respect to material permittivity, aims to efficiently optimize merit functions like this, where device performance is phrased in terms of electric and magnetic fields in an observation region^3–6^. Here, the electric field intensity at the center of each quadrant is maximized for correct wavelengths and polarizations, and minimized for incorrect ones through choice of sign in the *κ*(*q*, *p*, *λ*) weighting function where *p* indexes the linear polarization and *q* indexes the quadrant with center **r**_*q*_. The first summation targets broadband performance by including closely spaced wavelengths in each band to effectively optimize the device across a continuum. The purpose of the softplus function, *S*, is described in the methods alongside other optimization figures of merit for this work (Materials, methods, and additional text are available in the Supplementary section). This optimization function is nonlinear over the high-dimensional (~10^4^-dimensional for devices in this work) permittivity tensor, composed of deeply subwavelength volumetric units (voxels). It is optimized via gradient descent enabled by the well-known adjoint method^3,7,8^. Combining the electric fields in the device from adjoint simulations with those from the expected illumination, in this case broadband linearly polarized plane waves, the gradient is computed in a fixed number of simulations independent of the number of design voxels. Fabrication constraints were incorporated for layering, feature size control, and binarization using averaging, lateral maximum blurring, and sigmoid projection filters, respectively^28^.

**Fig. 2:**
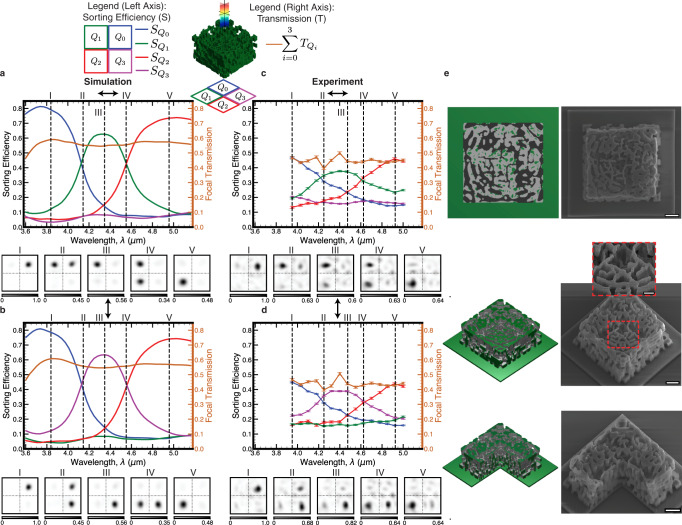
Fabrication and measurement results of multispectral and linear polarization sorting device. **a** (top) Simulated device sorting spectrum showing both relative sorting efficiency (*S*) and net transmission (*T*) to focal plane normalized to pinhole transmission. For quadrant *k*, ${S}_{{Q}_{k}}=\frac{{T}_{{Q}_{k}}}{\mathop{\sum }\nolimits_{i=0}^{3}{T}_{{Q}_{i}}}$. (bottom) Intensity images accounting for the expected imaging lens numerical aperture (NA = 0.67) and showing the focal spot moving as a function of wavelength. Each numbered plot corresponds to the labeled dashed vertical line in the spectrum above it. Intensity units are arbitrary, but comparable between all plots in (**a**). Different maximum values on the colorbars here and in other figures are labeled and utilized for better visibility of the plotted intensity features. **b** Same plots as in (**a**) for vertical polarization input. **c**, **d** Experimental comparison plots to (**a**, **b**) respectively with standard deviation (SD) error bars. Wavelength coverage differences between simulation and experiment are due to the limited tuning range of the QCL used experimentally. **e** Schematic and associated SEM images of fabricated devices. The rightmost device was printed with one quarter missing to show internal structure. Scale bars: 5 μm, 5 μm (inset 2 μm), 5 μm from top to bottom.

The optimization results are shown in Fig. 2a, b, where three sorting bands are present with the middle band focal spot conditioned on linear polarization. Following this result, the device was fabricated using the Nanoscribe Photonic Professional GT, where subwavelength features in the mid-infrared are readily created in a proprietary IP-Dip polymer with low loss from roughly 3.5–5.5 μm^29^. The real and imaginary refractive indices of this polymer averaged over the design wavelength range are accounted for in the device optimization and presented simulation results. Using a photolithography-based liftoff procedure, a series of 30 μm diameter circular aluminum apertures were fabricated on a sapphire substrate. Apertures, also included in the optimization, restrict the illumination to single devices for imaging and experimental power calibration. The Nanoscribe was aligned to write devices directly on top of the apertures. Figure 2e shows scanning electron microscope (SEM) images of fabricated devices. Each design was illuminated by a quantum cascade laser (QCL) with a beam waist on the order of the device size defocused such that the apertures were overfilled and sampled a roughly flat amplitude and phase section of the diverging beam. This is intended to mimic the plane wave input used for device optimization. The QCL can be tuned spectrally to probe the device at different wavelengths and the addition of linear polarizers and waveplates were used to control the input polarization. Various focal planes of the device were imaged by a zinc selenide (ZnSe) aspheric lens onto a focal plane array (see Fig. S1). The QCL used in the experiment had a limited wavelength tuning range from 3.95 to 5.05 μm, which is why the plots in Fig. 2c, d do not cover the full simulated spectra.

Figure 2c, d contains the experimental spectral and polarization sorting efficiency, overall focal transmission, and focal spot intensities to compare to simulation. Sorting efficiency measures the ratio of total focal plane signal incident on a given quadrant. In the simulated sorting efficiency, the middle band under horizontal (vertical) polarization has a width of 420 nm (430 nm) with a center wavelength of 4.34 μm (4.35 μm) with respect to its crossover points with the upper and lower bands. By comparison, in the experimental sorting efficiency, the middle band under horizontal (vertical) polarization has a width of 390 nm (420 nm) with a center wavelength of 4.42 μm (4.43 μm) with respect to its crossover points with the upper and lower bands, exhibiting a 20 nm (4.71%) smaller average width and an 80 nm (1.84%) average redshift. Taking into account this redshift and considering data over an equivalent total bandwidth, the peak sorting efficiencies for the three bands under horizontally (vertically) polarized illumination were 0.78, 0.63, 0.73 (0.78, 0.63, 0.74) in simulation and 0.47, 0.38, 0.46 (0.45, 0.39, 0.44) experimentally for lower, middle, and upper bands, respectively.

We suspect the reduced contrast in experiment is due to imaging aberrations, experimental beam non-idealities and fabrication errors, which include device shrinkage and feature size mismatch from proximity effects and resolution limits^30^. Transmission is measured as power through the device printed on top of a 30 μm aperture that reaches the focal plane versus power through an empty 30 μm aperture. We speculate the fluctuations in the experimental transmission around 4.25 μm and 4.4 μm could be due to minor laser power fluctuations around its transition between two QCL modules, small beam shifts between the device and pinhole normalization measurements, or differing amounts of ambient carbon dioxide (CO_2_) absorption between measurements given the long path optical path length and strong CO_2_ absorption near 4.25 μm^31^. In the focal plane images in Fig. 2c, d, one can see the focused spot move between the quadrants as the wavelength changes demonstrating the splitting functionality with the middle band sorted to opposite corners depending on its linear polarization.

### Full stokes polarimetry

For the second application, we investigated full Stokes imaging polarimetry, where one characterizes not only the linear polarization amplitudes, but also the phase relationship between them and the degree of polarization. This rich information is widely applicable, including in areas of biomedical imaging and diagnosis^32^, depth-based imaging and facial recognition^33,34^, atmospheric monitoring^35^, and bio-inspired polarization based navigation^36^. In polarimetric imaging, the input state is cast in terms of a four-dimensional vector containing its Stokes parameters, which together specify the orientation, handedness, and degree of polarization. Complete reconstruction of this state is done through at least four independent measurements. Measurements can be multiplexed in time using a rotating waveplate^37^ or in space by dividing up the area on one or more focal plane arrays^38^. The analogous geometry to using absorptive filters for color imaging is the division of focal plane (DoFP) technique where pixels are grouped together with each responsible for analyzing a specific polarization component. Many implementations use micropolarizer elements as filters^39^, thus limiting the transmission efficiency of the camera to 50% by rejecting orthogonally polarized light to each filter. Lost transmission can be recovered using pixel-sized metasurface lenses that apply different phase masks to two orthogonal polarizations. For example, six projections done pairwise onto orthogonal polarization basis states directly measure the four Stokes parameters^40^. However, these six measurements contain redundant information which reduces camera resolution or degrades signal-to-noise ratio compared to a four-measurement device with the same overall size. Recently, it was shown that a metasurface grating could project incident light onto four equally spaced analyzer states with each projection belonging to a different order^41^. This approach requires propagation to spatially separate each order and is inherently chromatic due to grating dispersion. We adopted benefits and addressed shortcomings of both approaches by employing the modularity of a pixel-level design for adaptation of any camera sensor to full polarimetric imaging and utilizing a minimal four-state projection for maximal compactness. Using only four measurements is a 33% improvement in required chip area or, alternatively, a commensurate resolution or signal-to-noise ratio enhancement. As an added benefit, inverse design provides a path towards broadband polarimetry, which is difficult to achieve with metasurface and waveplate based systems due to their inherent chromatic dispersion.

**Fig. 3:**
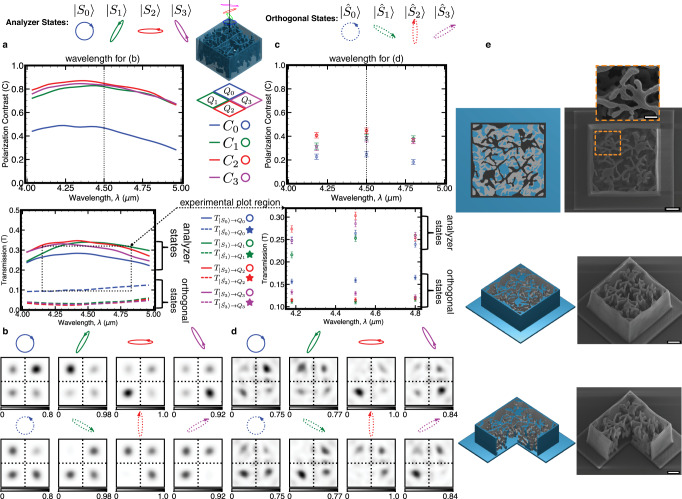
Fabrication and measurement results of Stokes polarimetry device. **a** (top) Polarization contrast (*C*) in simulation quantifying the transmission (*T*) into the desired quadrant for a given analyzer state versus transmission into the same quadrant for the orthogonal state. For input *k*, ${C}_{k}=\frac{{T}_{\left.|{S}_{k}\right\rangle \to {Q}_{k}}-{T}_{\left.|\hat{{S}_{k}}\right\rangle \to {Q}_{k}}}{{T}_{\left.|{S}_{k}\right\rangle \to {Q}_{k}}+{T}_{\left.|\hat{{S}_{k}}\right\rangle \to {Q}_{k}}}$. (bottom) Transmission into the desired quadrants for the analyzer states (solid) and their orthogonal complements (dashed). **b** Simulated focal intensity images (*λ* = 4.5 μm) accounting for imaging lens numerical aperture (NA =0.67) for the various input states where the top row contains analyzer states and the bottom row contains orthogonal states. Intensity units are arbitrary, but comparable between all plots in (**b**). **c** Comparison plots of contrast and transmission for the experimental results with analyzer states shown with open circles and orthogonal states shown with stars in the transmission plot (SD error bars). The experimental transmission plot region is different than the simulation one in both the *x*- and *y*-axes. This region is marked with a dashed box on the simulation plot in (**a**). **d** Experimental focal intensity images (*λ* = 4.5 μm) showing a bright quadrant for each analyzer state and the same quadrant dark for the complementary orthogonal state. Intensity units are arbitrary, but comparable between all plots in (**d**). **e** Schematic and associated SEM images of fabricated devices. The rightmost device was printed with one quarter missing to show internal structure. Scale bars: 5 μm (inset 2 μm), 5 μm, 5 μm from top to bottom.

We optimized a device of size 30 μm × 30 μm × 18 μm in six 3 μm layers for this purpose, with the optimization figure of merit adapted to focus four analyzer polarization states to different quadrants and reject their orthogonal states to those same quadrants. Further, we augmented the experimental system to probe arbitrary polarization states for different wavelengths depicted in Figs. S1, S2. The simulation and experimental results are presented in Fig. 3a–d and fabricated devices are shown in Fig. 3e. Performance is quantified with two metrics. First, for each quadrant, the contrast, *C* ∈ [ − 1, 1], is the transmission for an analyzer state versus its orthogonal state: $C=\frac{{T}_{{{{{{{{\rm{analyzer}}}}}}}}}-{T}_{{{{{{{{\rm{orthogonal}}}}}}}}}}{{T}_{{{{{{{{\rm{analyzer}}}}}}}}}+{T}_{{{{{{{{\rm{orthogonal}}}}}}}}}}$. The optimization solution performs better for the three elliptical polarizations compared to the circular polarization state in this case, likely due to a lack of degrees of freedom. In Supplementary Fig. S3, we show a thicker 12-layer device in simulation with improved contrast of the circular polarization state. Similar to the multispectral device, there is a reduced contrast experimentally which we attribute again to fabrication and experimental imperfections. Second, transmission is quantified for each analyzer state, which, as shown in the Polarimetry Contrast Bounds Supplementary section, is limited to 50% in a perfect device due to required vector overlaps between analyzer states^42^. We note that this is not a limit on total device transmission, but simply a requirement of linearity. Observing the focal plane images in Fig. 3b, d demonstrates the polarization sorting capability of the device. The most telling indication of desired behavior is seen by observing the orthogonal state inputs where the device can theoretically fully extinguish transmission to a quadrant (Materials, methods, and additional text are available in the Supplementary section). By comparison to the analyzer state, the same quadrant under each orthogonal state is dark, which is supported quantitatively with specific transmission and contrast values in Fig. 3a, c. Due to experimental and fabrication non-idealities, the measured device exhibits lower splitting contrast compared to simulation. For example, at *λ* = 4.5 μm, the simulated device achieved contrasts of 0.46, 0.82, 0.84, and 0.83 for analyzer states *S*_0_, *S*_1_, *S*_2_, and *S*_3_, respectively. In comparison, for these same four states at *λ* = 4.5 μm, the measured device achieved contrasts of 0.25, 0.39, 0.45, and 0.37. Practically, reconstruction of an arbitrary input polarization state can be done via a calibration procedure to account for imperfect contrast. An example calibration considering the simulated device behavior is demonstrated in the supplementary and analysis of reconstruction accuracy of pure and mixed polarization states is shown in Figs. S9 and S10, respectively (Materials, methods, and additional text are available in the Supplementary section).

#### Angular momentum sorting

**Fig. 4:**
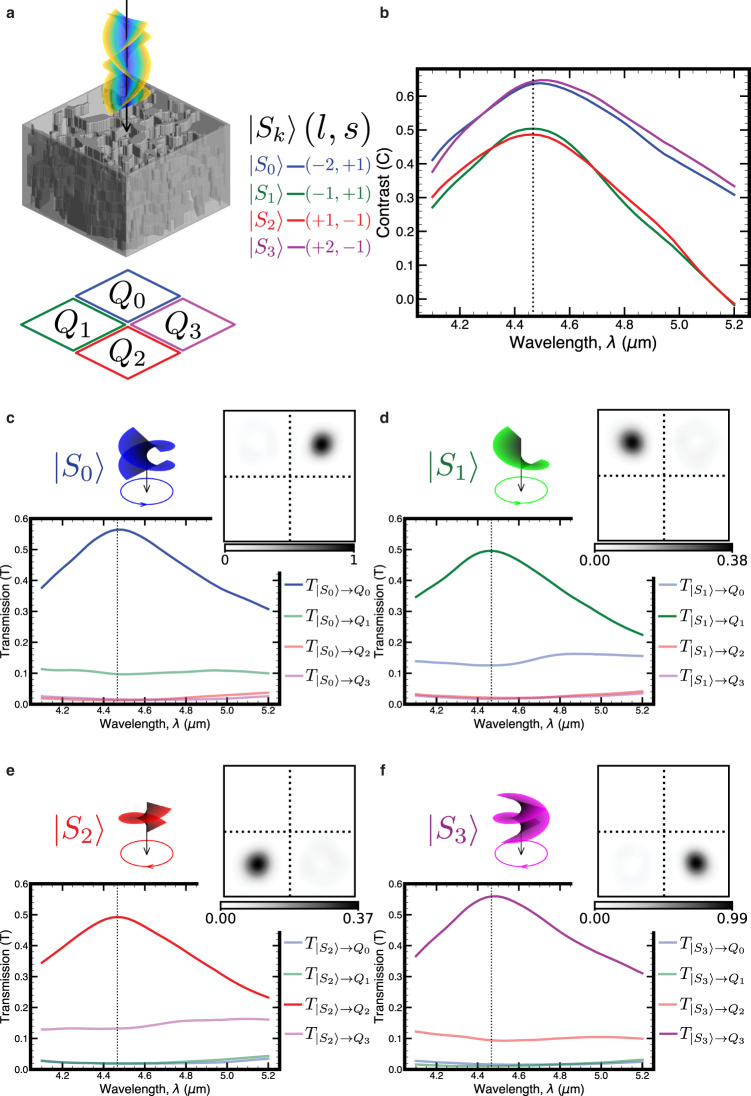
Simulation performance for angular momentum sorting device. **a** Schematic of device and focal plane quadrants. **b** Contrast for sorting each state (*C* ∈ [ − 1, 1]) defined by the transmission of a state into the desired quadrant versus the transmission into the rest of the focal plane. For source *k*, ${C}_{k}=\frac{{T}_{\left.|{S}_{k}\right\rangle \to {Q}_{k}}-{\sum }_{i\ne k}{T}_{\left.|{S}_{k}\right\rangle \to {Q}_{i}}}{{T}_{\left.|{S}_{k}\right\rangle \to {Q}_{k}}+{\sum }_{i\ne k}{T}_{\left.|{S}_{k}\right\rangle \to {Q}_{i}}}$. **c** Transmission spectrum for (*l* = − 2, *s* = + 1) input with desired quadrant transmission in blue. Transmission is normalized by power through the device aperture with no device present. Inset: Intensity at focal plane for wavelength marked with dashed vertical line (arbitrary units, but comparable to other intensity plots in figure). **d**–**f** Same plots as (**c**), but for (*l* = − 1, *s* = + 1), (*l* = + 1, *s* = − 1), (*l* = + 2, *s* = − 1), respectively.

A third device that we explored only in simulation sorts on the spatial degree of freedom. One property of wavefronts with spatial structure is their orbital angular momentum (OAM). Beams with discrete OAM values are modeled as Laguerre-Gaussian (LG) modes, which comprise a set of orthogonal spatial modes in the paraxial wave equation^43^ (Materials, methods, and additional text are available in the Supplementary section). These modes are candidates for free space optical communication networks where information can be multiplexed on both the OAM and spin degrees of freedom^44^. Isolated devices that efficiently demultiplex different angular momentum values in free space^45^ or from fibers^46^ are essential to high bandwidth communication links. Further, an efficient sorting device can also be used to reciprocally generate different OAM states when specific quadrants are illuminated in the focal plane. Additional communication bandwidth is achievable with further spatial multiplexing of angular momentum beamlets^47^, where the receiver requires an array of devices with similar geometry to those shown in this manuscript. In addition, spatially resolved OAM information can enhance contrast in imaging systems due to the asymmetric phase with azimuthal angle^48^ and can find applications in high bandwidth holographic optical encryption systems^49^. Applicable to either the isolated or array geometry, we consider a routing structure sensitive to combinations of four OAM states and two spins in the form of circular polarization handedness. Figure 4a illustrates the optimized angular momentum sorting device, consisting of 8 design layers, each 2.4 μm thick and a 30.15 μm × 30.15 μm lateral aperture. Sorting contrast by input *k*, *C*_*k*_ ∈ [ − 1, 1], is shown in Fig. 4b with an average value of 0.57 over the four angular momentum states at wavelength *λ* = 4.47 μm. Contrast is defined as transmission into the desired quadrant versus elsewhere in the focal plane for source *S*_*k*_ and target quadrant *Q*_*k*_, specifically ${C}_{k}=\frac{{T}_{{Q}_{k}}-{\sum }_{i\ne k}{T}_{{Q}_{i}}}{{T}_{{Q}_{k}}+{\sum }_{i\ne k}{T}_{{Q}_{i}}}$. Each combination of OAM and spin is efficiently focused to a different quadrant as seen in Fig. 4c–f. Transmission values for a beam splitting and subsequent filtering scheme as opposed to routing would be limited to 25% for each state, so the proposed device roughly doubles the signal-to-noise ratio of detection. The response of the device to excitations with OAM and spin values different from the design points is analyzed in Figs. S11–12.

## Discussion

In this work, we demonstrate multilayer, inverse-designed nanophotonic structures capable of augmenting both the performance and multifunctionality of imaging systems. Using the same configuration of lenses inside a typical camera and replacing the scattering element on top of the focal plane array, this technology enables cameras to be sensitive to angular momentum, polarization state, arbitrary spectral signatures, or combinations of multiple electromagnetic properties. There are exciting avenues for exploration in the mid-infrared where fabrication is accessible via TPP tools such as the Nanoscribe. We envision targeting specific narrow absorption bands for applications in chemical and biomedical imaging and tiling different types of splitting elements in the same array. Moving forward, we can think of these elements as part of a computational imaging system where we design efficient reconstruction problems by utilizing direct control over the scattering properties of an array of elements in the optical path^50^.

By utilizing a well-optimized fabrication procedure and additional design rules, TPP fabrication can be pushed to the near-infrared range^16^. Scaling to longer wavelengths in the infrared requires a polymer transparent beyond 5.5 μm or the use of a material inversion technique^51^ and parallel writing strategies for feasible fabrication times^52^. Currently, necessity of industry-level fabrication procedures are a barrier to demonstrating volumetric inverse design for visible wavelengths. Typical integrated circuits, like those found in modern computer processors, consist of greater than ten layers of precisely aligned subwavelength structures with respect to visible wavelengths^53^. By replacing metals with transparent optical materials in silicon-based CMOS processes, these fabrication techniques can realize the types of structures shown in this work at an industrial scale. Currently, cost, complexity, and availability of these fabrication methods limit the exploration of multilayer photonic devices in academic and prototype settings. Advances in accessible multilayer fabrication is a worthwhile endeavor to unlock a broad spectrum of imaging applications^54^. Beyond replacing traditional absorptive-based Bayer filters with color routing structures, optimized devices targeting structural color will impact reflective display technology^55^ and efficient, spectrally-selective waveguide couplers will improve performance of augmented reality displays^56^. We believe there is large, untapped potential for 3D, inverse-designed photonics in both research and commercial settings. The present work is a substantial step towards the realization of these complex devices for real-world applications.

## Methods

### Simulation

Simulations and inverse design optimizations of structures were carried out using Lumerical (ANSYS, Inc.) finite-difference time-domain (FDTD) Maxwell equations solver. Working in the time domain with pulsed sources, the broadband response of the device to forward and adjoint sources can be computed in single simulations. During design, we used a total field scattered field (TFSF) source to create a finite-sized, normally incident plane wave input to the device, depicted in Fig. S4. The simulation boundary conditions were perfectly matched layers (PML) to create the effect of an infinite simulation domain for the isolated structure.

When verifying and reporting simulation performance in the manuscript, we doubled the mesh resolution in the device region, increased the simulation mesh accuracy everywhere from 2 to 4 (with 1 being the least accurate and 8 considered the most accurate), and used a defocused Gaussian source that matched more closely to the experiment compared to the plane wave optimization source. We used a Gaussian beam with a waist radius of *w*_0_ = 12.5 μm and a defocus amount at the aperture opening of *z* = 500 μm such that the beam was diverging. For the angular momentum device which we did not validate experimentally, we used focused Laguerre-Gaussian modes as defined below with their waist positioned at the device face for both optimization and evaluation. We simulated devices on top of ${{{{{{{{\rm{A{l}}}}}}}_{2}{{{\rm{O}}}}}}}_{{{{{{{{\rm{3}}}}}}}}}$ substrates using material permittivity values from literature^57^. Further, for the multispectral and Stokes polarimetry we included the 30*μ*m diameter aperture in the optimization and evaluation using a perfect electrical conductor (PEC).

### Simulation resources

Device optimizations are run on a high performance computing cluster. During each optimization iteration, multiple forward and adjoint simulations are run to compute gradient information for the multi-objective problems specified below. For reference, we used 10 computing nodes for the multispectral and Stokes polarimetry devices and 12 computing nodes for the angular momentum device. Each node was allocated 8 Intel CPU cores (mix of Skylake 2.1 GHz and Cascadelake 2.2 GHz processors), to run simulations in parallel. With this, the optimizations complete in ~30–40 h of compute time depending on the specific device thickness in the paper. The thicker Stokes polarimetry device shown in the supplemental took roughly 78 h to complete.

### Optimization figures of merit and weighting

#### Multispectral and linear polarization

2 $$\begin{array}{r}\mathop{\max }\limits_{{{{{{{{\boldsymbol{\epsilon }}}}}}}}\in {\{{\epsilon }_{\min },{\epsilon }_{\max }\}}^{N}}g({{{{{{{\bf{E}}}}}}}})=\mathop{\sum}\limits_{\lambda }S\left(\left(\mathop{\sum}\limits_{p}\mathop{\sum}\limits_{q}\kappa (q,p,\lambda )\frac{{I}_{p}({{{{{{{{\bf{r}}}}}}}}}_{q},\lambda )}{{I}_{\max }(\lambda )}\right);k\right)\\ {I}_{p}({{{{{{{{\bf{r}}}}}}}}}_{q},\lambda )=||{{{{{{{{\bf{E}}}}}}}}}_{p}({{{{{{{{\bf{r}}}}}}}}}_{q},\lambda )|{|}^{2}\end{array}$$

where $S(x;k)=\frac{{{{{{{{\rm{\ln }}}}}}}}(1+{e}^{kx})}{k}$ is the softplus function, which ensures positivity of all figures of merit. For large *x*, which corresponds to focusing of a given wavelength primarily in the correct quadrant, this function acts in a linear regime with the onset of this regime controlled by the value *k*. In the opposite extreme, when a given wavelength is primarily in the undesired quadrants, *x* is negative and the figure of merit tapers to zero ensuring its gradient contribution is large in the dynamic performance weighting scheme. We use *k* = 2 for this optimization.

The polarization index, *p*, separates performance for plane wave excitations with different linear polarizations. The weighting *κ*(*q*, *p*, *λ*) directs bands of wavelengths evenly spaced between 3.7–5 μm to different quadrants, *q*, with the middle band sent to one of two locations based on linear polarization and the other two bands operating independent of polarization. Wavelengths outside of a given band for a target quadrant are punished through a negative weight using *κ*(*q*, *p*, *λ*).

3 $$\kappa (q,p,\lambda )=\left\{\begin{array}{ll}1,\quad &{{{{{{{\rm{if}}}}}}}}(\lambda,p)\,{{{{{{{\rm{desired}}}}}}}}\,{{{{{{{\rm{for}}}}}}}}\,{{{{{{{\rm{quadrant}}}}}}}}\,q\hfill \\ -\alpha,\quad &{{{{{{{\rm{if}}}}}}}}\,\lambda\, {{{{{{{\rm{within}}}}}}}}{{\,\Delta }}\lambda=\beta \frac{{{\Lambda }}}{3}{{{{{{{\rm{of}}}}}}}}\,{{{{{{{\rm{quadrant}}}}}}}}\,{q}^{{\prime} }{{{{{{{\rm{s}}}}}}}}\,{{{{{{{\rm{target}}}}}}}}\,{{{{{{{\rm{band}}}}}}}}\\ 0,\quad &{{{{{{{\rm{otherwise}}}}}}}}\hfill \end{array}\right.$$

Here, Λ is the full bandwidth of the optimization, in our case 1.3 μm. For our optimization, we used *β* = 0.5, which controls how far spectrally into the neighboring bands we explicitly reject intensity in a given quadrant. Finally, *α* sets the punishment weighting term for out-of-band light versus desired in-band light. We used *α* = 0.75.

The normalization term ${I}_{\max }(\lambda )$ accounts for the fact that for a fixed power, the intensity at the center of a focal spot will scale with wavelength. We use the scaling ${I}_{\max }(\lambda ;l,f)={l}^{2}/({f}^{2}{\lambda }^{2})$ for device lateral size, *l*, and focal length, *f*.

#### Full stokes polarimetry

In the Stokes polarimetry device, we searched for devices with high contrast in intensity in each quadrant when illuminated with an analyzer state versus the state orthogonal to it. For each analyzer state (*a*) and its orthogonal state ($\bar{a}$), we measured the intensity in the middle of the quadrant:

4 $${g}_{a,q} \, 	=\, \frac{{I}_{a,q}({{{{{{{{\bf{r}}}}}}}}}_{q},\lambda )}{{I}_{\max }(\lambda )}\\ {g}_{\bar{a},q} \, 	=\, \frac{{I}_{\bar{a},q}({{{{{{{{\bf{r}}}}}}}}}_{q},\lambda )}{{I}_{\max }(\lambda )}\\ {I}_{a,q} \, 	=\,||{{{{{{{{\bf{E}}}}}}}}}_{a,q}({{{{{{{{\bf{r}}}}}}}}}_{q},\lambda )|{|}^{2}\\ {I}_{\bar{a},q} \, 	=\,||{{{{{{{{\bf{E}}}}}}}}}_{\bar{a},q}({{{{{{{{\bf{r}}}}}}}}}_{q},\lambda )|{|}^{2}$$

We would like the analyzer value to be large and the other orthogonal value to be small, so we combined them together with the following product figure of merit:

5 $${g}_{q}(\lambda )={g}_{a,q}(\lambda )*(1-{g}_{\bar{a},q}(\lambda ))$$

Further, given the bound of 50% transmission for an analyzer state to a quadrant for a perfect device, we only optimized the parallel intensity up until that point. The net figure of merit, then, is:

6 $$\mathop{\max }\limits_{{{{{{{{\boldsymbol{\epsilon }}}}}}}}\in {\{{\epsilon }_{\min },{\epsilon }_{\max }\}}^{N}}g({{{{{{{\bf{E}}}}}}}})=\mathop{\sum}\limits_{\lambda }\mathop{\sum}\limits_{q}{g}_{q}(\lambda )$$

#### Angular momentum

7 $$\begin{array}{r}\mathop{\max }\limits_{{{{{{{{\boldsymbol{\epsilon }}}}}}}}\in {\{{\epsilon }_{\min },{\epsilon }_{\max }\}}^{N}}g({{{{{{{\bf{E}}}}}}}})=\mathop{\sum}\limits_{q}\mathop{\sum}\limits_{s}\mathop{\sum}\limits_{\lambda }\kappa (q,s)\frac{{I}_{s}({{{{{{{{\bf{r}}}}}}}}}_{q},\lambda )}{{I}_{\max }(\lambda )}\\ {I}_{s}({{{{{{{{\bf{r}}}}}}}}}_{q},\lambda )=||{{{{{{{{\bf{E}}}}}}}}}_{s}({{{{{{{{\bf{r}}}}}}}}}_{q},\lambda )|{|}^{2}\end{array}$$

The weighting function *κ*(*q*, *s*) controls whether focusing into a given quadrant, *q*, is desirable for a given mode source *s*. For a total number of optimization iterations, *M*, we define *κ*(*q*, *s*; *m*) parameterized by iteration number, *m*, as

8 $$\begin{array}{r}\kappa (q,s;m)=\left\{\begin{array}{ll}1,\hfill &{{{{{{{\rm{if}}}}}}}}\,q=s\hfill \\ -{w}_{{{{{{{{\rm{end}}}}}}}}},\hfill &{{{{{{{\rm{if}}}}}}}}\,m\ge {m}_{{{{{{{{\rm{end}}}}}}}}},\, q\, \ne \, s\\ -\left({w}_{{{{{{{{\rm{start}}}}}}}}}+({w}_{{{{{{{{\rm{end}}}}}}}}}-{w}_{{{{{{{{\rm{start}}}}}}}}})\frac{m}{{m}_{{{{{{{{\rm{end}}}}}}}}}-1}\right),\quad &{{{{{{{\rm{if}}}}}}}}\,m < {m}_{{{{{{{{\rm{end}}}}}}}}},\, q\, \ne \, s\end{array}\right.\end{array}$$

We chose ${w}_{{{{{{{{\rm{start}}}}}}}}}=\frac{1}{3}$, *w*_end_ = 1, *m*_end_ = 90, and *M* = 300. The first case corresponds to one quadrant being excited for a given source, so it receives a positive weight. The second two cases describe a linear ramp of negative weight for rejecting intensity into incorrect quadrants for a certain number of iterations after which point a constant negative weight is used for the rest of the optimization. With the dynamic performance weighting function described below, it is important that individual figures of merit stay positive for the entire optimization. The ramping of the negative weighting helps in this regard and our specific optimization maintains positive individual figures of merit throughout. We emphasize this is not a guarantee of the weighting scheme above, but a fortunate instance where it worked for our optimization. In other optimizations, we explicitly ensured the individual figures of merit remained positive.

#### Dynamic weighting

All optimizations are multi-objective and require balancing many individual figures of merit. One way of achieving a balance and preventing certain figures of merit from dominating the optimization solution is by using a dynamic performance-based weighting scheme. As certain figures of merit start performing better than others, their relative optimization weight decreases. For *N*_*FOM*_ individual figures of merit, the *j*^*th*^ figure of merit with current performance *f*_*j*_ (with net performance defined as ∑_*j*_*f*_*j*_) is weighted:

9 $${w}_{j}=\frac{2}{{N}_{{{{{{{{\rm{FOM}}}}}}}}}}-\frac{{f}_{j}^{p}}{{\sum }_{n}{f}_{n}^{p}}$$

For the multispectral and linear polarization optimization, *f*_*j*_ corresponded to each wavelength figure of merit. In other words, ${f}_{j}={f}_{\lambda }=S(({\sum }_{p}{\sum }_{q}\kappa (q,p,\lambda )\frac{{I}_{p}({{{{{{{{\bf{r}}}}}}}}}_{q},\lambda )}{{I}_{\max }(\lambda )});k)$. In the Stokes polarimetry case, *f*_*j*_ corresponded to each product figure of merit for combinations of quadrant and wavelength. Specifically, each *g*_*q*_(*λ*) was an individual figure of merit in the weighting scheme. Finally, for the angular momentum optimization, each quadrant figure of merit corresponded to an *f*_*j*_. In other words, ${f}_{j}={f}_{q}={\sum }_{s}{\sum }_{\lambda }\kappa (q,s)\frac{{I}_{s}({{{{{{{{\bf{r}}}}}}}}}_{q},\lambda )}{{I}_{\max }(\lambda )}$.

We chose *p* = 2 for all optimizations. This weighting scheme relies on positive individual figures of merit to function properly. With the above formula, weights can become negative when a given figure of merit is far ahead of the others. In these cases, we simply shifted and rescaled to ensure all weights were ≥0. After computing individual gradients for each figure of merit, $\frac{\partial {f}_{j}}{\partial \overrightarrow{\epsilon }}$, the net gradient was computed using this weighting scheme as ${\sum }_{j}{w}_{j}\frac{\partial {f}_{j}}{\partial \overrightarrow{\epsilon }}$.

The gradients derived during the optimization were based on a focusing figure of merit (norm electric field squared at a point) and use dipole excitations as adjoint sources. Typically, this is a good proxy for transmission into a given quadrant, which is ultimately what we care to achieve with devices placed on top of focal plane arrays. For some optimizations, the transmission-measured performance is used instead of the intensity-measured performance as the input to the dynamic weighting function. This reduced dependencies on exact normalizations of intensity with power and took into account electric field profiles that looked different than a traditional focusing profile or were shifted from the center of the quadrant.

### Optimization fabrication constraints

Projection filters were used to push the optimization solution towards devices that respected certain fabrication constraints^28^. These filters are differentiable functions applied in sequence to a design variable in order to create a device variable. The device variable describes the structure being optimized and that will eventually be fabricated. We found the gradient of the figure of merit with respect to the device variable and then backpropagated that gradient to the design variable using the chain rule. The design variable was then stepped in the gradient direction.

For binarization, we used a sigmoid filter of the form $f({\rho }_{k})=\frac{\tanh (\beta \eta )+\tanh (\beta ({\rho }_{k}-\eta ))}{\tanh (\beta \eta )+\tanh (\beta (1-\eta ))}$. The strength, *β*, was increased over a series of 10 epochs, each with 30 iterations, starting at *β* = 0.0625 and doubling each epoch. The center point was fixed at *η* = 0.5. For layering, permittivity values were averaged vertically over the layer thickness at each lateral point. This corresponds to averaging the two-dimensional gradient of each slice in a given layer during backpropagation.

For minimum feature size, we used an averaging blurring function that tapered from the center pixel, specifically:

10 $$f({\rho }_{k})=\frac{1}{N}\mathop{\sum}\limits_{{r}_{j}\in {{{\Omega }}}_{k}}\frac{{b}_{r}+1-{r}_{j}}{{b}_{r}}{\rho }_{j}$$

where $N={\sum }_{{r}_{j}\in {{{\Omega }}}_{k}}\frac{{b}_{r}+1-{r}_{j}}{{b}_{r}}$ for normalization^58^. *b*_*r*_ is the blur radius based on a circle inscribed on a square with sides equal to the desired minimum feature size of 750 nm, such that ${b}_{r}=0.5*\sqrt{2}*750{{{{{{{\rm{nm}}}}}}}}=530{{{{{{{\rm{nm}}}}}}}}$ (*r*_*j*_ is the distance of voxel *j* from voxel *k*). We computed this sum out to ${r}_{j,\max }={b}_{r}$. This filter tends to increase the density value of voxels nearby a solid one. This encourages a minimum feature size in the solid domain. However, the drawback of this method is that it does not guarantee a minimum feature size and we do end up with features smaller than the minimum value in our final designs. This can be improved through filters that blur even further out, directly fixing the design grid to be that of minimum feature size increments, or use of a level set procedure at the end of the optimization with a feature size constraint. The other disadvantage of this method is it only attempts to control feature size in the solid domain and does not address minimum gap sizes in the void domain.

Some fabrication constraints are difficult to encapsulate in a filtering function. Final designs needed to consist of a single piece of material for fabrication. Further, with the Nanoscribe, an enclosed void cannot be realized because the liquid polymer would have no way to escape during development. During the optimization, every 8 iterations, each design layer was patched to ensure it was a single piece of material and bridges formed in this step were restricted from changing for the next 8 iterations until the patching occurred again. The bridges were chosen via a shortest path (Dijkstra’s) graph algorithm with the cost equal to the amount a given voxel would need to move to become fully solid. Islands of material were connected via a greedy minimum spanning tree approach with net bridge costs used as the edge weight for connecting two islands together. The density value of the inserted bridge was then set to 0.75 (fully solid corresponds to a density of 1) everywhere along its path. This works to ensure solid connectivity and often the void connectivity is maintained by chance throughout the optimization. Final patches were done after the optimization to create void connectivity if it had not happened naturally. These were usually minimal changes that did not have large effects on the device performance. Nevertheless, the reported simulation results in this manuscript used the fully patched designs that were fabricated.

### General optimization details

For each design, the pre-filtered density is initialized to a midpoint uniform value of 0.5 (*ρ* ∈ [0, 1]). For some designs, additional built-in structural robustness is added by enforcing a solid material border around the edges of the device. This has little effect on the final optimization result. Only one initial seed is used for each optimization and results tend to not be plagued by poor-performing local minima. Empirically, when optimizing with a relatively low index contrast as is the case in this work, designs tend to converge to well-performing solutions without needing to run large numbers of individual optimizations. However, augmenting the methods presented here to perform more of a global search on the design space could potentially result in even higher performance. The number of layers was fixed for each design and chosen empirically to achieve high photonic performance while still maintaining fabricability. Further, the layer height was not part of the optimization process. All layers were optimized simultaneously where each layer gradient was computed as the average gradient over all of the vertical slices in the layer. While not shown here via direct comparison to single layer designs, the necessity of multilayer structures for these types of sorting tasks has been evaluated in the past including our prior work on this topic^12^. Further, Fig. S3 demonstrates the utility of adding thickness via additional layers to a device optimization region. It shows improvement of the sorting contrast for circular polarization in the polarimetry device.

### Device fabrication

The fabrication procedure for the measured devices is shown in Fig. S5. Devices were printed directly on top of 30 μm apertures defined via a photolithography-based lifotff procedure. Apertures were 150 nm thick and controlled the illumination on the device. They were also used for measuring beam power through blank apertures to normalize net transmission of the device. Negative-tone photoresist, AZ nLoF 2070 (MicroChemicals GmbH), was patterned with photolithography to create a variety of apertures as well as alignment marks for the optical setup. Following oxygen and argon direct plasma cleaning to remove undesired residual photoresist left after development, 150 nm of aluminum (Al) was deposited via electron beam evaporation. The apertures were lifted off in acetone and the substrate was cleaned in IPA followed by DI water. IP-Dip resist was dropped onto the substrate surface for direct write lithography using the Nanoscribe Photonic Professional GT. In the Nanoscribe, the apertures were located by moving the stage after the substrate surface was found by the microscope. By turning on the laser below the polymerization threshold such that the microscope could still image a fluorescence signal from the laser focus, we aligned the center of the printing axes to the aperture center. After writing, the devices were developed in propylene glycol methyl ether acetate (PGMEA) for 20 min and rinsed in two successive IPA baths for 3 min each. The surface of the substrate was dried with a gentle nitrogen stream. We found that critical point drying was not necessary for the integrity of our structures through the drying process. For imaging in the scanning electron microscope, a 5nm coating of platinum (Pt) was sputtered onto the surface to reduce charging effects due to the insulating polymer.

### Optical experimental setup

The optical setup shown in Fig. S1 illuminated the devices through the sapphire substrate with a diverging Gaussian beam across the pinhole aperture on which the device was printed. Polarization in the case of the multispectral device was changed via a half-wave plate and linear polarizer where the former served to rotate more power into the polarization less overlapped with the laser output mode. In the case of the Stokes measurement setup, a combination of a linear polarizer, half-wave plate and quarter-wave plate were used to achieve desired input states to the device. These wave plates applied a wavelength-dependent retardance, which was taken into account in their chosen rotation. To ensure the polarization states were correct, we used a traditional method for reconstruction of the polarization state consisting of a quarter-wave plate followed by a Wollaston prism (polarizing beam splitter). The prism split orthogonal linear polarizations into two different angles which were imaged onto a power meter. By rotating the quarter-wave plate to three known rotations and measuring the power in each angle, we reconstructed what the input polarization state must have been. The reconstructed state overlaps are shown in Fig. S2c for each probing wavelength of the Stokes measurement setup.

#### Imaging, focal length, and chromatic dispersion

The imaging objective, L2 in Fig. S1, was translated in the axial direction to measure different planes moving back from the substrate surface. First, we observed and verified in simulation the presence of significant focal shift with respect to the focal length of the device with wavelength due to chromatic aberration of the aspheric lens. Modeling the optical setup in simulation using the Stellar Software Beam4 open source ray tracing program, we found this shift to be 31.4 μm between *λ* = 3.95 μm and *λ* = 5 μm. Experimentally, we measured this effect by imaging the diffraction pattern of an empty 30 μm aperture on the substrate surface. By tuning the focus until the diffraction pattern disappeared, we could ensure we were focused on the aperture surface for a given wavelength. We adjusted the axial position of the lens for different laser wavelengths until we were at the surface of the aperture and noted the micrometer position in order to characterize the chromatic focal shift of the imaging lens. Experimentally, we computed this dispersion to be 46*μ*m for the same range of *λ* = 3.95 μm and *λ* = 5 μm. If we assume this can be used as a calibration of the micrometer on the stage, then each marking corresponds to 0.68 μm tick^−1^.

Using stage markings, we found the best focal plane of the multispectral device for *λ* = 3.95 μm to be located at 63 ticks from the substrate surface. For a total device height of 19.5 μm and designed focal length of 25 μm, we expected the focus to be located at 44.5 μm off the substrate surface. Applying the calibration above of 0.68 μm tick^−1^, we estimate the measured focal plane to be located at 43 μm from the surface (*f* = 23.5 μm for assumed device height of 19.5 μm), which is close to the design and within reasonable inaccuracies of the above calibration and small errors in printed device height.

For the multispectral device, we took 15 measurements evenly spaced between 3.95 μm and 5 μm. Since the chromatic dispersion is not equal across this whole range, we broke the range into two parts and linearly interpolated the axial position of the imaging objective to probe the same focal plane for each wavelength. Between 3.95 μm and 4.48 μm, we interpolated over 26 ticks corresponding to 17.7 μm and between 4.48 μm and 5 μm, we interpolated over 20 ticks corresponding to 13.7 μm.

For the Stokes polarimetry device, we measured at three distinct wavelengths, 4.18, 4.5, and 4.8 μm. We directly set the focal lengths based on the empirical axial position of a blank 30 μm aperture on the substrate for each wavelength. We used the same focal length of 63 ticks corresponding to around 43 μm. The device height in this case was designed to be 19.8 μm, so this corresponded to *f* = 23.2 μm.

#### Transmission normalization

The imaging of device focal planes onto the camera needed to be calibrated with a net device transmission. We quantified the transmission of the device by using an empty circular aperture on the substrate with the same diameter as the one sectioning off illumination to the device. Using a power meter, we measured the power through the aperture without the device versus the power through the aperture with the device. We tracked the beam center on the camera and the method depended on properly centering the beam on both apertures. Further, we assumed that laser power was not fluctuating significantly in time and the beam center was not shifting upon successive wavelength tuning due to thermal effects and a changing laser mode. Using this method, we saw consistent and expected transmission values through the device with only minor fluctuations for the multispectral device around 4.4 μm, which may have been due to invalidation of assumptions previously stated. This wavelength is close to the crossover between two modules in the QCL covering different spectral ranges and we speculate the power may be less stable here compared to other wavelengths. The measured transmission was assumed to be contained in the camera image of the focal plane and its surrounding area. We then assumed that the transmission corresponding to a patch of the camera image was equal to the ratio of its intensity to the total intensity multiplied by the net measured transmission. Transmission into the focal plane, for example, was computed by multiplying the measured total transmission value by the ratio of intensity in the focal plane to the intensity in the focal plane and surrounding area. Camera images were taken of the focal plane for each wavelength and a background of the camera (with the laser emission off) was taken immediately afterward. Taking the background immediately after each measurement reduced error in the background drifting over the course of the long experimental procedure from temperature drifts in the room or the camera housing itself. These background images were subtracted from the camera images.

### Stokes state creation and verification

Each polarization state was generated through choice of rotation of a linear polarizer, a half-wave plate, and a quarter-wave plate pictured in Fig. S2a. The wave plates are chromatic components with a retardance defined for a given wavelength. Using the provided retardance data from Thorlabs for each component, we computed the effect a rotated component will have on each input wavelength. By optimizing the choice of angles of these three components, we could generate all of the desired input polarization states to the device. To verify the correctness of each state, we used the setup in Fig. S2b consisting of a quarter-wave plate and Wollaston prism. The Wollaston prism splits the input polarization into its x- and y-polarization components, each of which were imaged onto and measured by a power meter. Under different rotations of the quarter-wave plate, the magnitude of x- and y-polarized components will change as a function of the input state. By measuring these components under three rotations and using the specified retardance values of this quarter-wave plate as a function of wavelength, we reconstructed the input state. We used rotation values of 0^∘^, 22^∘^, and 44^∘^ of the fast axis with respect to the x-polarization direction. Plotted in Fig. S2c are the magnitudes of the vector overlaps of the reconstructed and the desired Jones state for each wavelength. Note the y-axis on the plot begins at 0.9.

## Supplementary information

Supplementary Information

Peer Review File

## Data Availability

Data to support the conclusions in the manuscript can be provided on request.
