# Supplementary material for: 3D-patterned inverse-designed mid-infrared metaoptics
Source: Nat Commun. 2023 May 13;14:2768. doi: 10.1038/s41467-023-38258-2 (PMC10183040; doi:10.1038/s41467-023-38258-2)
Supplement: Supplementary file 1 — Supplementary Information [file 41467_2023_38258_MOESM1_ESM.pdf]

3D-Patterned Inverse-Designed Mid-Infrared Metaoptics Supplementary

Contents

1 Two-Photon Polymerization (TPP) Accuracy **2**

2 Laguerre Gaussian Modes for Angular Momentum Splitter **2**

3 12-Layer Stokes Polarimetry Device **2**

4 Polarimetry Splitting Bounds **3**
- 4.1 Maximum transmission into each analyzer state 3
- 4.2 Minimum overlap between analyzer states 4

5 Polarimetry Contrast Bounds **5**
- 5.1 Analyzer state transmission to all quadrants 5
- 5.2 Extinguishing orthogonal state to analyzer quadrant 6

6 Polarimetry Analyzer States **6**

7 Device Index of Refraction Profiles **7**

8 Polarimetry Reconstruction **7**
- 8.1 Reconstruction Method 8
- 8.2 Reconstructing Pure Polarization States 9
- 8.3 Reconstructing Mixed Polarization States 9

9 Angular Momentum Sorting Device Outside of Design Points **9**
- 9.1 Illumination with Different Spin Values 10
- 9.2 Illumination with Different OAM Values 10

10 Supplementary Figures **11**
- 10.1 Optical setup for characterization of multispectral and polarimetry devices 11
- 10.2 Stokes state creation and verification 11
- 10.3 Simulation performance for Stokes polarimetry device with additional degrees of freedom compared to Stokes polarimetry device from the main text 13
- 10.4 Schematic of simulation geometry for optimization and evaluation . . 15
- 10.5 Schematic of fabrication process 16
- 10.6 Conceptual diagram of the Stokes polarimetry device 16
- 10.7 Index of refraction profiles for multispectral and angular momentum devices 17
- 10.8 Index of refraction profiles for Stokes polarimetry devices 19
- 10.9 Stokes polarimetry reconstruction for pure states 21
- 10.10 Stokes polarimetry reconstruction for mixed states 23

10.11 Angular momentum device under different spin excitation 24
10.12 Angular momentum device under different OAM excitation 26

References **28**

1 Two-Photon Polymerization (TPP) Accuracy

Fabrication via TPP is a flexible and powerful method, but also has known challenges in printing accuracy [1]. We observe shrinkage of the structure, which is dependent on the height of the layer from the substrate. Material printed on the bottom layer is not able to shrink from its printed size because it is physically adhered to the substrate. The topmost layer is roughly 90% of the desired lateral size and the bottom layer is close to the expected size. We also observe dilation of the smallest features in the design. Designs were compensated for this effect by pre-eroding features in the STL file before printing. Finally, the Nanoscribe had a mismatch between the feature size in each lateral direction. This is not a limitation of TPP, but likely the result of astigmatism in the optical alignment of our specific tool.

2 Laguerre Gaussian Modes for Angular Momentum Splitter

A spatially varying field can carry orbital angular momentum (OAM). Discrete values of OAM, l, can be found in the Laguerre-Gaussian orthonormal basis for solutions of the paraxial wave equation [2]. We used a simplified set with $p = 0$, such that each mode was defined at its waist ($z = 0$) with spatial profile in cylindrical coordinates:

$$u(r, \phi, z = 0) = (\frac{r\sqrt{2}}{w_0})^{|l|} e^{\frac{-r^2}{w_0^2}} e^{-il\phi} \tag{1}$$

where w_0 is the waist radius of the beam. We chose $w_0 = 8.5\mu\text{m}$ to ensure the mode was confined to the device. Transmission plots shown are geometrically normalized against the transmission of this beam through the device aperture with no device present. We can further assign a spin angular momentum of the mode by choosing the handedness of its circular polarization. The following pairs of OAM values l and spin values s were used in the optimization: $(l, s) = (-2, 1), (-1, 1), (1, -1), (2, -1)$. These states were assigned to quadrants starting with the top right (blue) and moving counterclockwise (green, red, magenta).

3 12-Layer Stokes Polarimetry Device

The polarimetry device in the main text consists of six $3\mu\text{m}$ layers and struggles to achieve equal contrast for all four analyzer states with the circular polarization state lagging the others. We speculate this may be due to lack of degrees of freedom in the thickness of device. As a comparison, we optimize a thicker device consisting of twelve $3\mu\text{m}$ layers to see if the solution will display better contrast for all analyzer

states. In `Fig. S3` we show the comparison of the thicker device to the original. While the quadrant transmission per analyzer state is slightly reduced, the contrast metric is improved for the circular polarization state without sacrificing the other analyzer state contrasts.

4 Polarimetry Splitting Bounds

We can model the Stokes polarimetry device as a linear system that projects an input Jones state describing the x- and y-polarized electric field components onto several analyzer states. The Jones polarization is a 2-dimensional complex vector. The four analyzer states for our device are specifically chosen Jones vectors. In `Fig. S6`, analyzer states correspond to $|v_i\rangle$, where $N = 4$ for the device in the paper. We assume the device outputs into four spatially distinct modes $|w_k\rangle$, such that we take them to be orthogonal ($\langle w_i|w_k\rangle = \delta_{ik}$). Specifically, we model each output mode as a focused spot in a different quadrant of the focal plane and thus we assume the lack of spatial overlap implies orthogonality to a good approximation. The functionality of the device is described by an operator $\hat{Q}$ where projection of an input state on each analyzer direction modulates the amplitude of an outgoing mode. We write

$$\hat{Q} = \sum_i \alpha_i \left|w_i\right\rangle \left\langle v_i\right| \tag{2}$$

Without loss of generality, we assume α_i is real. Any complex phase can be included in output mode $|w_i\rangle$.

4.1 Maximum transmission into each analyzer state

Next, we assume for simplicity that all states have the same projection efficiency, such that $\alpha_i = \alpha$. The transmission bound will differ from the following if each state does not split at the same projection efficiency. Consider an arbitrary state $|a\rangle$ and it's orthogonal complement $|\bar{a}\rangle$. The action of $\hat{Q}$ on $|a\rangle$ is

$$\hat{Q}\left|a\right\rangle = \alpha \sum_i \left|w_i\right\rangle \left\langle v_i|a\right\rangle \tag{3}$$

Taking the vector magnitude squared of the resulting state

$$\langle a|\hat{Q}^\dagger \hat{Q}|a\rangle = \alpha^2 \sum_{i,j} \left\langle w_i|w_j\right\rangle \left\langle v_j|a\right\rangle \left\langle a|v_i\right\rangle \tag{4}$$

Since $\langle w_i|w_k\rangle = \delta_{ik}$, the double sum reduces to

$$\langle a|\hat{Q}^\dagger \hat{Q}|a\rangle = \alpha^2 \sum_i \left\langle v_i|a\right\rangle \left\langle a|v_i\right\rangle = \alpha^2 \sum_i |\left\langle a|v_i\right\rangle|^2 \tag{5}$$

Following this pattern, we also have

$$\langle \bar{a}|\hat{Q}^\dagger\hat{Q}|\bar{a}\rangle = \alpha^2 \sum_i \langle v_i|\bar{a}\rangle \langle \bar{a}|v_i\rangle = \alpha^2 \sum_i |\langle \bar{a}|v_i\rangle|^2 \tag{6}$$

Due to energy conservation, we cannot have gained any magnitude through applying $\hat{Q}$ on the state so $\langle a|\hat{Q}^\dagger\hat{Q}|a\rangle \leq 1$ and $\langle \bar{a}|\hat{Q}^\dagger\hat{Q}|\bar{a}\rangle \leq 1$. Summing these together, we get

$$\langle a|\hat{Q}^\dagger\hat{Q}|a\rangle + \langle \bar{a}|\hat{Q}^\dagger\hat{Q}|\bar{a}\rangle = \alpha^2 \sum_i (|\langle a|v_i\rangle|^2 + |\langle \bar{a}|v_i\rangle|^2) \leq 2 \tag{7}$$

Because the Jones vector space is 2-dimensional, $|a\rangle$ and $|\bar{a}\rangle$ form an orthonormal basis, so by definition $(|\langle a|v_i\rangle|^2 + |\langle \bar{a}|v_i\rangle|^2) = 1$. Thus, the sum simply becomes

$$\langle a|\hat{Q}^\dagger\hat{Q}|a\rangle + \langle \bar{a}|\hat{Q}^\dagger\hat{Q}|\bar{a}\rangle = N\alpha^2 \leq 2 \tag{8}$$

If we assume α is the largest it can be, then $\alpha^2 = \frac{2}{N}$. For $N = 4$ as is the case for the device in this manuscript, $\alpha^2 = 0.5$. Thus, the maximum transmission we can achieve for each analyzer state into its output mode is 0.5.

4.2 Minimum overlap between analyzer states

Given a maximum transmission efficiency of 0.5 for each analyzer state, we can set a minimum overlap, β, for Jones vector analyzer states used in the splitter. While the choice is not unique, a maximally spaced set of vectors will have a common mutual overlap. Assume for our set of analyzer states,

$$|\langle v_i|v_j\rangle|^2 = \begin{cases} 1 \text{ if } i = j \\ \beta^2 \text{ if } i \neq j \end{cases} \tag{9}$$

Sending in an analyzer state to the device

$$\hat{Q}\,|v_k\rangle = \alpha \sum_i |w_i\rangle \langle v_i|v_k\rangle \tag{10}$$

Taking the magnitude like before and using the orthogonality of the $|w_i\rangle$ states

$$\langle v_k|\hat{Q}^\dagger\hat{Q}|v_k\rangle = \alpha^2 \sum_i \langle v_i|v_k\rangle \langle v_k|v_i\rangle = \alpha^2 \sum_i |\langle v_k|v_i\rangle|^2 \tag{11}$$

Using the common overlap between states in the analyzer set and requiring that by energy conservation this magnitude squared is bound by 1,

$$\langle v_k|\hat{Q}^\dagger\hat{Q}|v_k\rangle = \alpha^2(1 + (N-1)\beta^2) \leq 1 \tag{12}$$

The relation between α and β, then is given by

$$\alpha^2 \leq \frac{1}{1+(N-1)\beta^2} \tag{13}$$

Suppose we specialize to the case where the transmission is maximized into each analyzer state ($\alpha^2 = \frac{2}{N}$) and we have no lost transmission for any given analyzer state through the system ($\langle v_k|\hat{Q}^\dagger\hat{Q}|v_k\rangle = 1$). Then,

$$\begin{aligned} \alpha^2(1+(N-1)\beta^2) &= 1 \\ \frac{2}{N}(1+(N-1)\beta^2) &= 1 \\ 1+(N-1)\beta^2 &= \frac{N}{2} \\ (N-1)\beta^2 &= \frac{N-2}{2} \\ \beta^2 &= \frac{N-2}{2(N-1)} \end{aligned} \tag{14}$$

Note the case of $N = 2$ requires no overlap between the vectors with $\beta^2 = 0$ and $\alpha^2 = \frac{2}{N} = 1$ because that matches the dimensionality of the Jones vector space. However, from two measurements, we cannot reconstruct the full Stokes vector where in order to do so we need at least $N = 4$. As stated before, for $N = 4$, $\alpha^2 = 0.5$ at best and with no lost transmission for the analyzer states, $\beta^2 = \frac{1}{3}$.

5 Polarimetry Contrast Bounds

The contrast figure of merit for the Stokes polarimetry device is independent of overall transmission. For a given quadrant corresponding to analyzer state $|v_i\rangle$ and orthogonal complement $|\bar{v_i}\rangle$, the contrast is related to the analyzer transmission T_{analyzer} and orthogonal transmission $T_{\text{orthogonal}}$ to the quadrant as $C = \frac{T_{\text{analyzer}} - T_{\text{orthogonal}}}{T_{\text{analyzer}} + T_{\text{orthogonal}}}$. In order to get a contrast of $C = 1$, we need to be able to completely extinguish light in the analyzer quadrant for the orthogonal state.

5.1 Analyzer state transmission to all quadrants

We first show that a given analyzer state must necessarily appear in more than just the desired quadrant. Following from the notation above, the action of the device on an analyzer state, $|v_k\rangle$ is given by

$$\hat{Q}\,|v_k\rangle = \sum_i \alpha_i\,|w_i\rangle\,\langle v_i|v_k\rangle \tag{15}$$

We ask how much overlap does this have with one of the output modes $|w_j\rangle$ not corresponding to the analyzer quadrant (i.e. $i \neq j$).

$$\langle w_j|\hat{Q}|v_k\rangle = \sum_i \alpha_i \langle w_j|w_i\rangle \langle v_i|v_k\rangle = \alpha_j \langle v_j|v_k\rangle \tag{16}$$

where we used $\langle w_j|w_i\rangle = \delta_{ij}$ to eliminate the sum. However, as we showed above, with four analyzer states, $\langle v_j|v_k\rangle \neq 0$ even for $j \neq i$. So there is energy in the other quadrants according to the splitting efficiency of the jth analyzer state and the overlap between the j and k analyzer states.

5.2 Extinguishing orthogonal state to analyzer quadrant

We now check if an orthogonal state can be completely extinguished to the analyzer quadrant, which will determine if we can achieve a contrast of $C = 1$. When we send in the orthogonal state to a given analyzer, $|\bar{v_k}\rangle$, the device output is given by

$$\hat{Q}\,|\bar{v_k}\rangle = \sum_i \alpha_i\,|w_i\rangle \langle v_i|\bar{v_k}\rangle \tag{17}$$

Since it is true that $\langle v_k|\bar{v_k}\rangle = 0$ by definition, the sum is reduced to

$$\hat{Q}\,|\bar{v_k}\rangle = \sum_{i\neq k} \alpha_i\,|w_i\rangle \langle v_i|\bar{v_k}\rangle \tag{18}$$

Now, we ask how much overlap does this have with the output mode corresponding to this analyzer quadrant, $|w_k\rangle$, since we are interested in seeing if this overlap can be zero.

$$\langle w_k|\hat{Q}|\bar{v_k}\rangle = \sum_{i\neq k} \alpha_i \langle w_k|w_i\rangle \langle v_i|\bar{v_k}\rangle = 0 \tag{19}$$

where $\langle w_k|w_i\rangle = \delta_{ki}$ is only nonzero for $i = k$, but the sum explicitly ranges over values of $i \neq k$. Thus, we can extinguish a quadrant completely for a given orthogonal state and a contrast of 1 is theoretically achievable even if we transmit all incident light through the device to the focal plane.

6 Polarimetry Analyzer States

The choice of analyzer states that fits the above criteria is not unique, but will correspond to a tetrahedron with points lying on the Poincaré sphere. First, we choose evenly spaced pure polarization states in Stokes space and then evaluate their mutual overlaps in Jones space. One state is fixed in Stokes space to be right circular polarization (RCP), which is encoded as $\begin{bmatrix}1, & 0, & 0, & 1\end{bmatrix}$. This choice is arbitrary and different starting states will generate equally suitable sets of analyzer states. Staying on the Poincaré sphere surface means the first entry is fixed to 1 (from

here, we write the vector in terms of S_1, S_2, and S_3). The other three states should lie on a circle with a fixed polar angle from this first state such that all mutual overlaps are the same. For polar angle θ and azimuthal angle ϕ, these states can be parameterized $\left[\sin\theta\cos\phi,\ \sin\theta\sin\phi,\ \cos\theta\right]$. To evenly spread out these states azimuthally, the spacing should be $\Delta\phi = \frac{2\pi}{3}$. We make the non-unique choice to set the first $\phi = 0$. The first two states on the circle, then are $\left[\sin\theta,\ 0,\ \cos\theta\right]$ and $\left[\sin\theta\cos\frac{2\pi}{3},\ \sin\theta\sin\frac{2\pi}{3},\ \cos\theta\right]$. Evaluating the dot product between any of the states on the circle and the right circular polarization state yields $\cos\theta$. The first two states on the circle have a dot product of $\sin^2\theta\cos\frac{2\pi}{3} + \cos^2\theta$. Equating these two values generates the relation:

$$\sin^2\theta\cos\frac{2\pi}{3} + \cos^2\theta = \cos\theta \tag{20}$$

Solving for $\cos\theta$ gives $\cos\theta = -\frac{1}{3}$. Completing the tetrahedron, the final Stokes states (rounded to the thousands place) are:

$$\begin{aligned} \left[1,\ 0,\ 0,\ 1\right] \\ \left[1,\ -0.471,\ 0.816,\ -0.333\right] \\ \left[1,\ 0.943,\ 0,\ -0.333\right] \\ \left[1,\ -0.471,\ -0.816,\ -0.333\right] \end{aligned} \tag{21}$$

Converting these states to Jones vectors, the analyzer states we used (rounded to the thousands place) are given by:

$$\begin{aligned} \left[0.707,\ -0.707j\right] \\ \left[0.514,\ 0.794 + 0.324j\right] \\ \left[0.986,\ 0.169j\right] \\ \left[0.514, -0.794 + 0.324j\right] \end{aligned} \tag{22}$$

The squared overlap magnitudes between any of these states, $\beta^2 = \frac{1}{3}$ as desired for equally split analyzer states.

7 Device Index of Refraction Profiles

Optimized index of refraction profiles for the multispectral and angular momentum sorting devices are shown in `Fig. S7` and those for the Stokes polarimetry device from the main text and the one from the supplement with more layers are shown in `Fig. S8`.

8 Polarimetry Reconstruction

The following section shows how the polarimetry device presented in the main text can be used to recover the Stokes parameters of arbitrarily polarized inputs. This

addresses interpretation of quadrant outputs when the excitation is different than the four analyzer states used in the design. It further addresses the ability of the device to utilize the four measurements to recover the degree of polarization for partially polarized light. This exploration is done in simulation, but the same calibration and reconstruction procedure can be used experimentally as well.

8.1 Reconstruction Method

The problem of converting the signal in each of the four quadrants into the incident polarization state can be phrased as follows:

$$\underline{\underline{\mathbf{M}}}\mathbf{S} = \mathbf{T} \tag{23}$$

where $\underline{\underline{\mathbf{M}}}$ is the forward model that maps the Stokes vector, $\mathbf{S}$, to the observed quadrant transmissions, $\mathbf{T}$. We utilize the common definition of the Stokes parameters:

$$\mathbf{S} = \begin{bmatrix} S_0 \\ S_1 \\ S_2 \\ S_3 \end{bmatrix} = \begin{bmatrix} E_x^2 + E_y^2 = E_{45}^2 + E_{-45}^2 = E_R^2 + E_L^2 \\ E_x^2 - E_y^2 \\ E_{45}^2 - E_{-45}^2 \\ E_R^2 - E_L^2 \end{bmatrix} \tag{24}$$

where E_x, E_y, E_{45}, and E_{-45} are projections onto horizontal, vertical, 45-degree, -45-degree linear polarizations, respectively and E_R and E_L are projections onto right- and left-circular polarizations, respectively. To calibrate the device, we input each of these individual polarization components and observe the transmission into each of the four quadrants. Then, we form:

$$\begin{aligned} \underline{\underline{\mathbf{M}}}\underline{\underline{\boldsymbol{\sigma}}} &= \underline{\underline{\boldsymbol{\tau}}} \\ \underline{\underline{\boldsymbol{\sigma}}} &= \begin{bmatrix} \mathbf{S}_x & \mathbf{S}_y & \mathbf{S}_{45} & \mathbf{S}_{-45} & \mathbf{S}_R & \mathbf{S}_L \end{bmatrix} \in \mathbb{R}^{4x6} \\ \underline{\underline{\boldsymbol{\tau}}} &= \begin{bmatrix} \mathbf{T}_x & \mathbf{T}_y & \mathbf{T}_{45} & \mathbf{T}_{-45} & \mathbf{T}_R & \mathbf{T}_L \end{bmatrix} \in \mathbb{R}^{4x6} \\ \underline{\underline{\mathbf{M}}} &\in \mathbb{R}^{4x4} \end{aligned} \tag{25}$$

where $\mathbf{S}_x = \begin{bmatrix} 1 & 1 & 0 & 0 \end{bmatrix}^\dagger$, $\mathbf{S}_y = \begin{bmatrix} 1 & -1 & 0 & 0 \end{bmatrix}^\dagger$, $\mathbf{S}_{45} = \begin{bmatrix} 1 & 0 & 1 & 0 \end{bmatrix}^\dagger$, $\mathbf{S}_{-45} = \begin{bmatrix} 1 & 0 & -1 & 0 \end{bmatrix}^\dagger$, $\mathbf{S}_R = \begin{bmatrix} 1 & 0 & 0 & 1 \end{bmatrix}^\dagger$, $\mathbf{S}_L = \begin{bmatrix} 1 & 0 & 0 & -1 \end{bmatrix}^\dagger$ and $\mathbf{T}_\alpha$ are the four quadrant transmissions under excitation by the the $\mathbf{S}_\alpha$ state. We solve for $\underline{\underline{\mathbf{M}}}$ by taking the pseudo-inverse of $\underline{\underline{\boldsymbol{\sigma}}}$ and applying it on the right side, $\underline{\underline{\mathbf{M}}} = \underline{\underline{\boldsymbol{\tau}\boldsymbol{\sigma}}}^\dagger$. Then, we form the solution or reconstruction matrix by taking the inverse of $\underline{\underline{\mathbf{M}}}$, such that given a set of measurements $\mathbf{T}$, we compute the Stokes parameters as $\mathbf{S} = \underline{\underline{\mathbf{M}}}^{-1}\mathbf{T}$. We note this calibration could alternatively be done with the four analyzer states used in the design and we expect the results would be similar.

8.2 Reconstructing Pure Polarization States

The reconstruction method applied to pure polarization states is shown in `Fig. S9` for different amounts of added noise in the transmission measurements to simulate different signal-to-noise ratios in the sensor detection. For p added noise, we add a normally distributed random variable with a mean of 0 and a standard deviation equal to $p * T_{\text{avg}}$ where T_{avg} is the mean transmission across the four quadrant transmissions. As can be seen for increasing noise, the S_3 parameter is the most susceptible to a reduced signal-to-noise ratio. This is likely due to the circular polarization analyzer state exhibiting the lowest contrast and the S_3 Stokes parameter being a direct measure of the handedness of the circular polarization in the input.

8.3 Reconstructing Mixed Polarization States

The use of four projective measurements means information about partially polarized input states is contained in the quadrant transmissions. To test our ability to recover this property, we consider the situation where the polarization vector input into the device is randomly changing. We input a series of random polarization states into the device, and average the resulting quadrant transmission values for each quadrant. From these averaged transmission values, we reconstruct the Stokes vector in the same way as above. This reconstructed vector is compared to the averaged Stokes vectors for all the states input into the device. The degree of polarization of the light is computed as $p = \frac{\sqrt{S_1^2+S_2^2+S_3^2}}{S_0}$.

`Fig. S10` shows the results of reconstructing mixed polarization states. As the number of averaged states increases, the degree of polarization starts dropping. When noise is added per averaged state (using the same type of distribution as above), the squared error for the reconstruction is highest for the smaller number of averaged states. As this number of states increases, the fluctuating noise term starts averaging to zero thus decreasing the overall effect of noise on the reconstruction.

9 Angular Momentum Sorting Device Outside of Design Points

`Fig. S11` and `Fig. S12` demonstrate the behavior of the angular momentum sorting device for different values of spin and OAM, respectively, than the design states. In an optical communication application, controlling the behavior of the device at these alternate points will depend on the amount noise present and mode distortion between communication links. However, in an advanced imaging context where information about the scene is inferred through the spatially resolved projection of the input onto different angular momentum states, the response of the device to other mode inputs needs to be at least characterized if not explicitly designed for the given application. As a note, the optimization technique used here was not directed to explicitly minimize or control the behavior of the device under these other excitations. By adding more simulations to each iteration to capture the effect of illuminating with these other modes, we can compute a gradient that either

enables control over the quadrant these other modes couple to or extinguishes their transmission.

9.1 Illumination with Different Spin Values

In `Fig. S11`, we observe the device behaves similarly upon a flip in the handedness of the circular polarization for each angular momentum state. This can be seen through similar contrast and transmission profiles albeit at lower overall values. Thus, the optimization solution for the device relied primarily on the different OAM values for splitting and does not have strong polarization discriminating behavior.

9.2 Illumination with Different OAM Values

In `Fig. S12`, we observe the device output changes drastically when illuminated with different OAM values. Most of the light for each of the four states goes to the quadrants designed for the original higher design OAM values (i.e. - $l = -2, +2$). This is the reason for the negative contrast in the other two quadrants. Further, overall transmission values are significantly reduced with the higher transmission occurring for OAM values closer to the design points (i.e. - $l = -3, +3$).

10 Supplementary Figures

10.1 Optical setup for characterization of multispectral and polarimetry devices

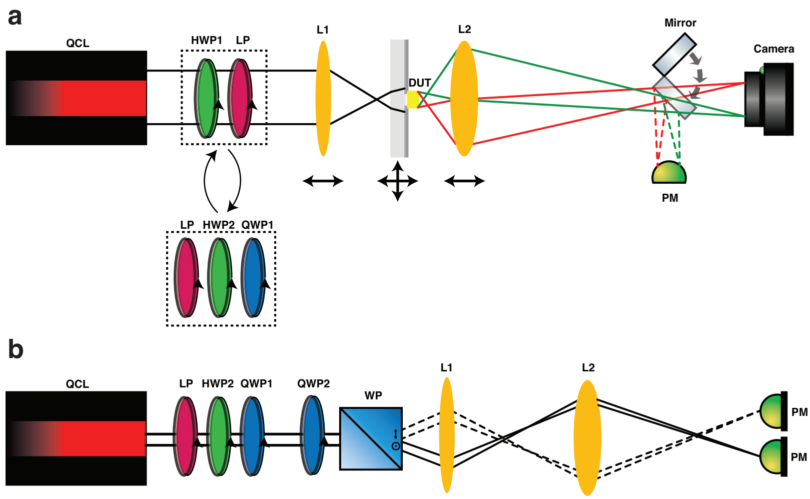

Supplementary Fig. 1: **(a)** Configuration for imaging of device focal plane and power normalization. Without the mirror in place, the lens images focal planes of the device onto the camera. Normalization of the device transmission is done with the mirror and power meter path of the setup. For these measurements, net power through an empty aperture is used to normalize net power through an aperture of the same size with the device on top of it. The power meter is aligned to the beam center, which is aligned to the pinhole centers during measurement. QCL: MIRcat-QT Mid-IR Quantum Cascade Laser (DRS Daylight Solutions); HWP1: Thorlabs WPLH05M-4500, Low-Order 4.5μm Half-Wave Plate; HWP2: Thorlabs WPLH05M-5300, Zero-Order 5.3μm Half-Wave Plate; QWP 1: Thorlabs WPLQ05M-4500, Low-Order 4.5μm Quarter-Wave Plate; LP: Thorlabs WP25M-IRA, Wire Grid Polarizer; L1: Thorlabs AL72525-E1, ZnSe Aspheric Lens, NA=0.42; L2: Thorlabs AL72512-E1, ZnSe Aspheric Lens, NA=0.67; Camera: Electrophysics PV320L IR Camera. **(b)** Configuration for verifying the polarization states used to test the Stokes polarimetry device. The second quarter-wave plate is moved to three distinct positions and the power in each linear polarization component separated by the Wollaston prism is recorded. QWP2: Thorlabs WPLQ05M-3500, Low-Order 3.5μm Quarter-Wave Plate; WP: Thorlabs WPM10, Wollaston Prism.

10.2 Stokes state creation and verification

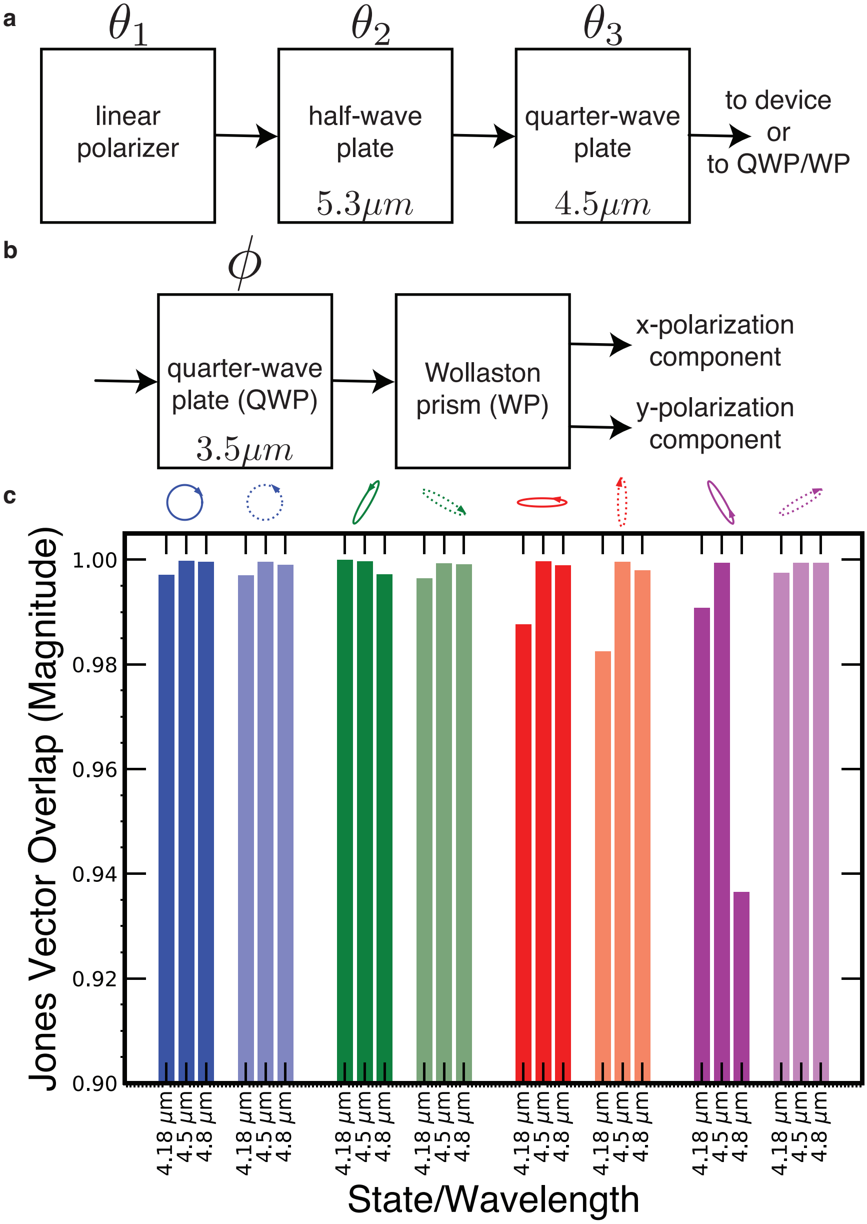

Supplementary Fig. 2: **(a)** Polarization states are created through choice of angles of the linear polarizer, half-wave plate, and quarter-wave plate (θ_1, θ_2, and θ_3). **(b)** Each state is verified by measuring the horizontal and vertical polarization component magnitudes output from the Wollaston prism after the state passes through a quarter-wave plate under three different rotations, ϕ. **(c)** Plot of measured Jones vector overlap for the 4 analyzer states and their 4 orthogonal complements for each measurement wavelength used in the experiment.

10.3 Simulation performance for Stokes polarimetry device with additional degrees of freedom compared to Stokes polarimetry device from the main text

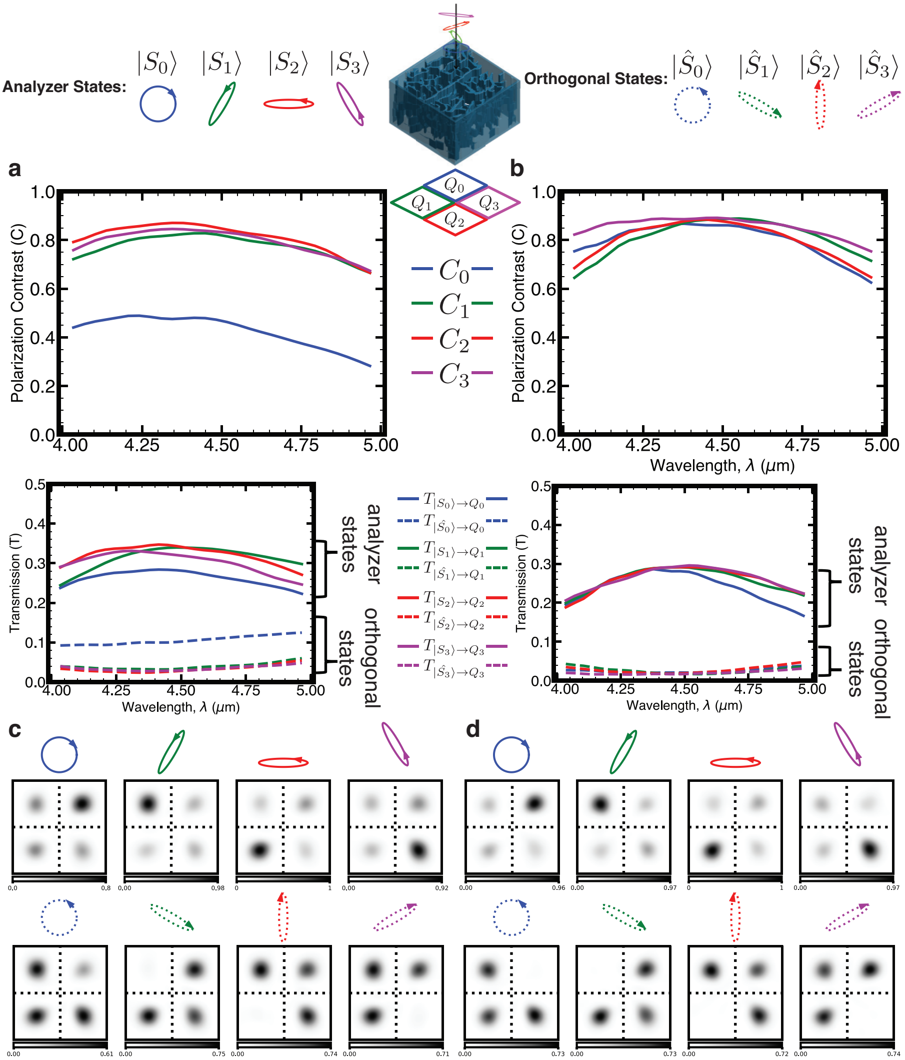

Supplementary Fig. 3: **(a)** Polarization contrast (C) and transmission (T) for device from the main text showing low contrast for the circular polarization analyzer state. For input k, $C_k = \frac{T_{|S_k\rangle\to Q_k} - T_{|\hat{S_k}\rangle\to Q_k}}{T_{|S_k\rangle\to Q_k} + T_{|\hat{S_k}\rangle\to Q_k}}$. **(b)** Polarization contrast and transmission for device with additional degrees of freedom showing high contrast for all four analyzer states at the cost of slightly reduced analyzer state transmission. **(c)** Focal intensity images for device from the main text with the top row showing the analyzer states and the bottom row showing their orthogonal complements. Intensity units are arbitrary but comparable between all plots in (c). The focal plane size is same as device aperture (30μm x 30μm). **(d)** Focal intensity image comparison for the device with additional degrees of freedom. Intensity units are arbitrary but comparable between all plots in (d). The focal plane size is the same as device aperture (30μm x 30μm).

10.4 Schematic of simulation geometry for optimization and evaluation

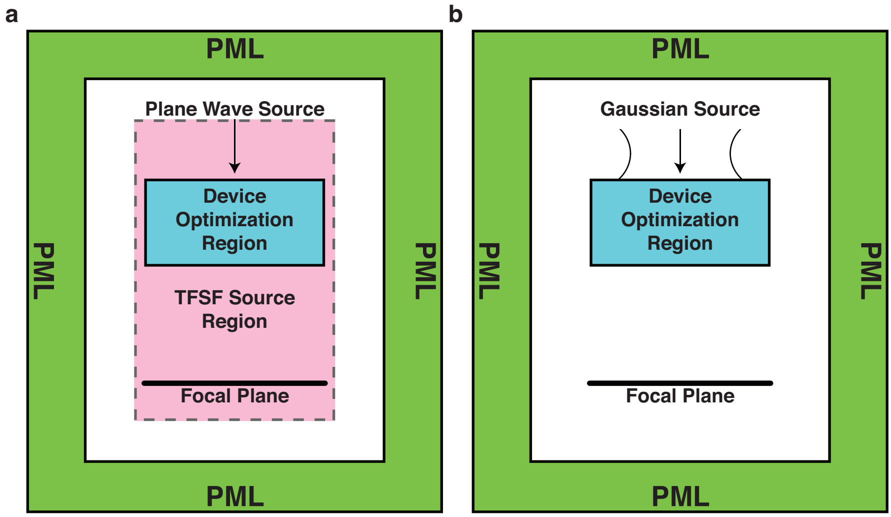

Supplementary Fig. 4: **(a)** Simulation geometry for optimization of the multispectral and Stokes polarimetry devices using a plane wave excitation. The angular momentum devices are optimized using focused angular momentum states with different circular polarization handedness for spin. **(b)** Evaluation geometry for the multispectral and Stokes polarimetry devices where the plane wave excitation is replaced with a defocused Gaussian source intended to match with the experimental source. Angular momentum devices are evaluated with the same sources as used for optimization.

10.5 Schematic of fabrication process

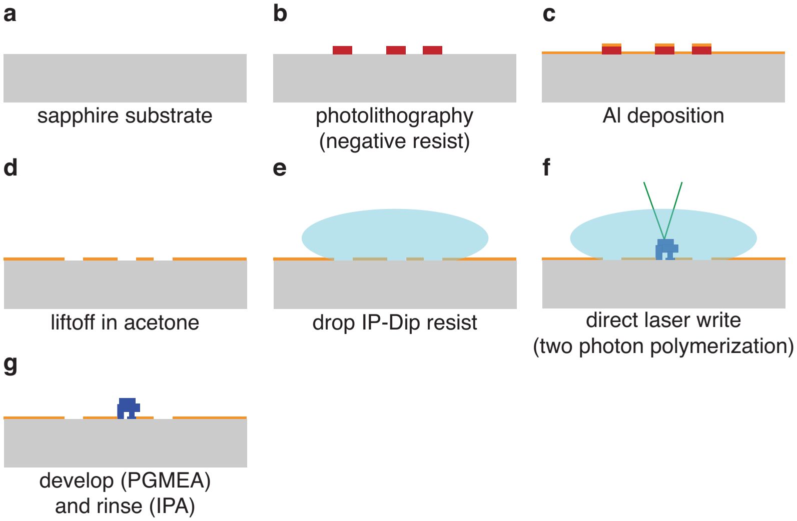

Supplementary Fig. 5: **(a)** Fabrication starts with a sapphire substrate (Al_2O_3, C-plane (0001), double side polished, 2-inch diameter, 0.5mm thickness). **(b)** Using a negative tone photoresist, apertures are patterned onto the substrate using photolithography. **(c)** After direct oxygen and argon plasma cleaning to remove undesired residual resist on the substrate, 150nm of Al is deposited on top using an electron beam evaporator. **(d)** The liftoff procedure is finished in acetone to remove remaining photoresist followed by cleaning in IPA and then DI water. **(e)** The IP-Dip resist from Nanoscribe is dropped onto the substrate. **(f)** Alignment is done by keeping the laser power below polymerization threshold and using fluorescence from its focused spot to align to the aperture centers for printing. **(g)** Development in propylene glycol methyl ether acetate (PGMEA) for 20 minutes followed by two three-minute rinses in IPA reveals the final device.

10.6 Conceptual diagram of the Stokes polarimetry device

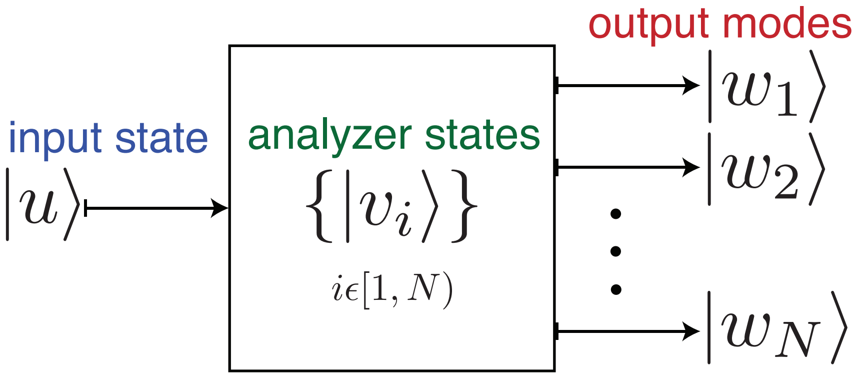

Supplementary Fig. 6: Conceptual diagram of the Stokes polarimetry device. The device acts to project an input state onto four outgoing states depending on its overlap with each analyzer state.

10.7 Index of refraction profiles for multispectral and angular momentum devices

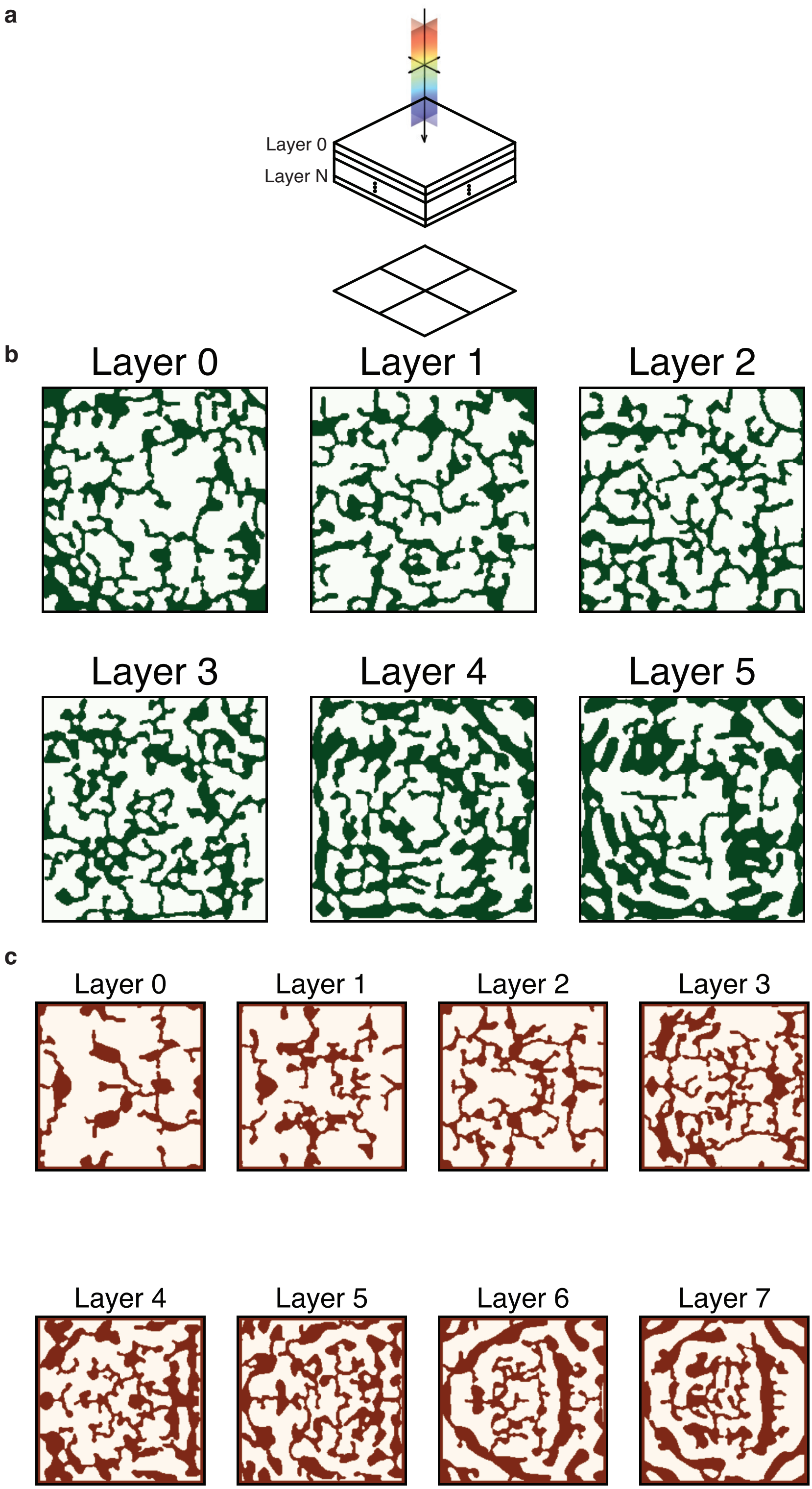
a
Layer 0
Layer N
b
Layer 0
Layer 1
Layer 2
Layer 3
Layer 4
Layer 5
c
Layer 0
Layer 1
Layer 2
Layer 3
Layer 4
Layer 5
Layer 6
Layer 7

Supplementary Fig. 7: Index of refraction profiles for multispectral (b) and angular momentum (c) devices. Dark colors are IP-Dip polymer and light areas are void. **(a)** Schematic of geometry showing the location of each labeled layer. Each layer is $3\mu m$ thick for the multispectral device and $2.4\mathrm{\mu m}$ thick for the angular momentum device. **(b)** Multispectral and linear polarization device index profile with each layer $30.15\mathrm{\mu m}$ x $30.15\mathrm{\mu m}$. **(d)** Angular momentum device index profile with each layer $30.15\mathrm{\mu m}$ x $30.15\mathrm{\mu m}$.

10.8 Index of refraction profiles for Stokes polarimetry devices

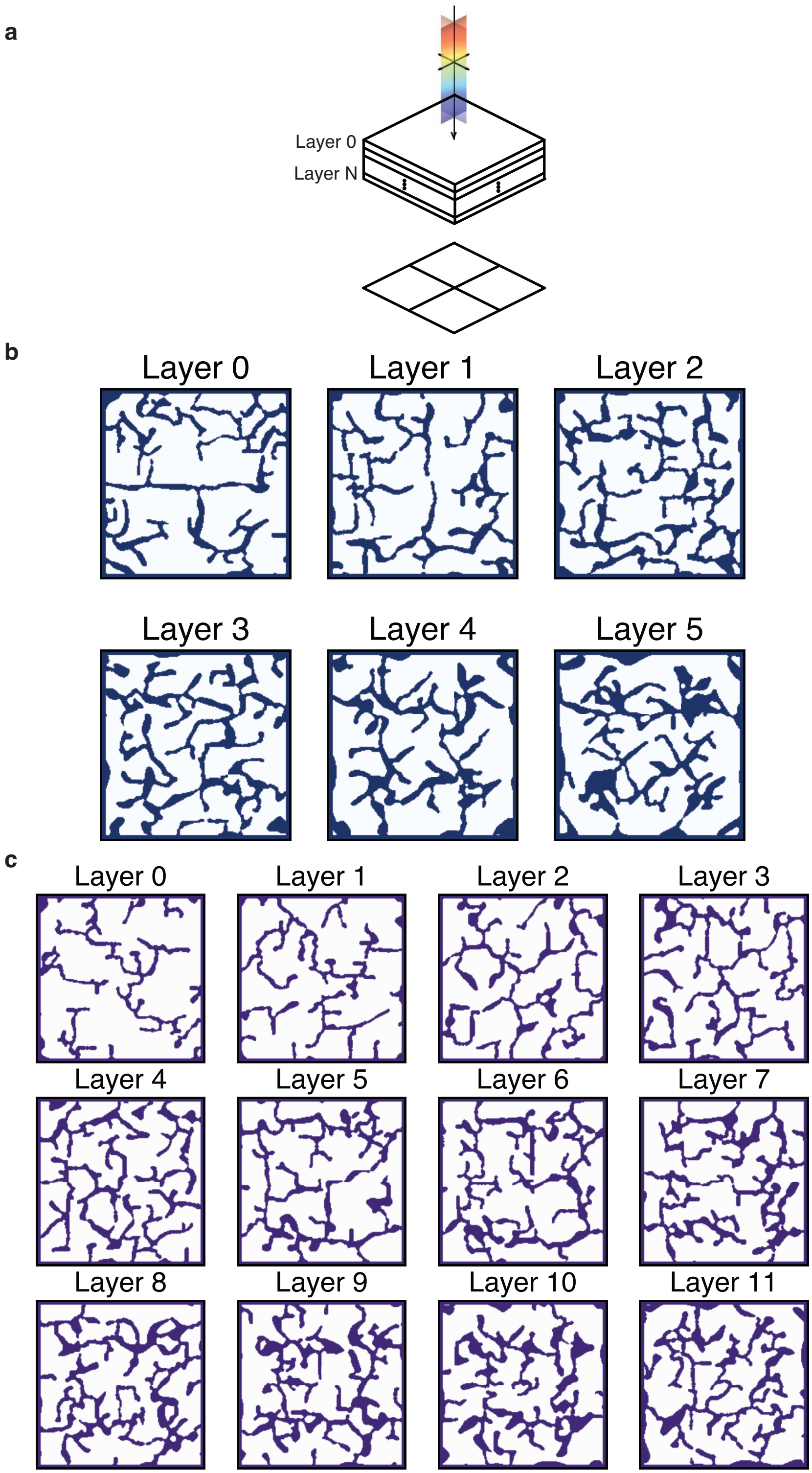
a
Layer 0
Layer N
b
Layer 0
Layer 1
Layer 2
Layer 3
Layer 4
Layer 5
c
Layer 0
Layer 1
Layer 2
Layer 3
Layer 4
Layer 5
Layer 6
Layer 7
Layer 8
Layer 9
Layer 10
Layer 11

Supplementary Fig. 8: Index of refraction profiles for Stokes polarimetry device from main text (b) and Stokes polarimetry device with more degrees of freedom from supplement (c). Dark colors are IP-Dip polymer and light areas are void. **(a)** Schematic of geometry showing location of each labeled layer. Each layer is $3\mu\text{m}$ thick. **(b)** Stokes polarimetry device from main text index profile with each layer $30\mu\text{m}$ x $30\mu\text{m}$. **(c)** Stokes polarimetry device with additional degrees of freedom index profile with each layer $30\mu\text{m}$ x $30\mu\text{m}$.

10.9 Stokes polarimetry reconstruction for pure states

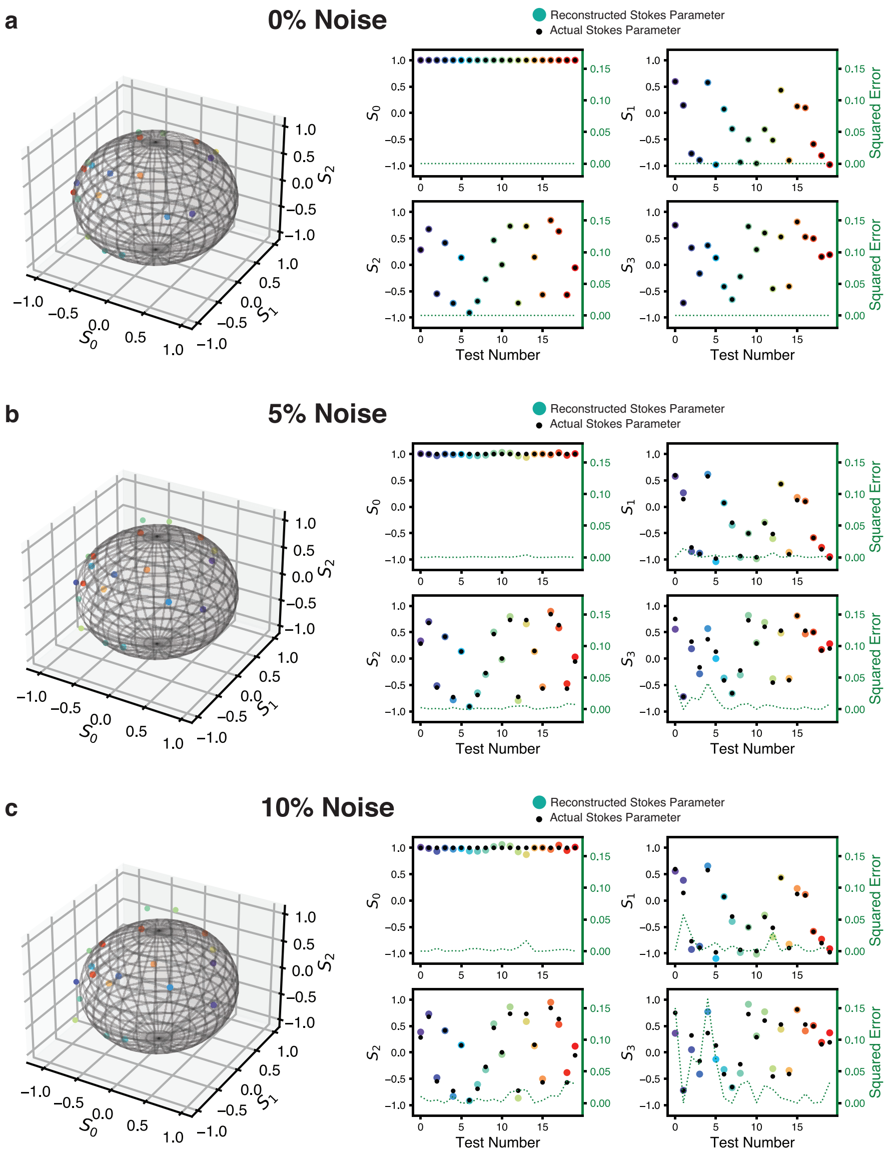

Supplementary Fig. 9: Stokes polarimetry reconstruction in simulation using the device from the main text under random pure polarization inputs at a wavelength of $4.5\mu\mathrm{m}$. **(a)** Reconstructed state locations shown on Poincaré sphere on the left and comparison of the reconstructed Stokes parameters to the actual ones shown on the right with the associated squared error (green dashed line, right y-axis). **(b)** Same plots as (a) but with 5% added noise. **(c)** Same plots as (a) but with 10% added noise.

10.10 Stokes polarimetry reconstruction for mixed states

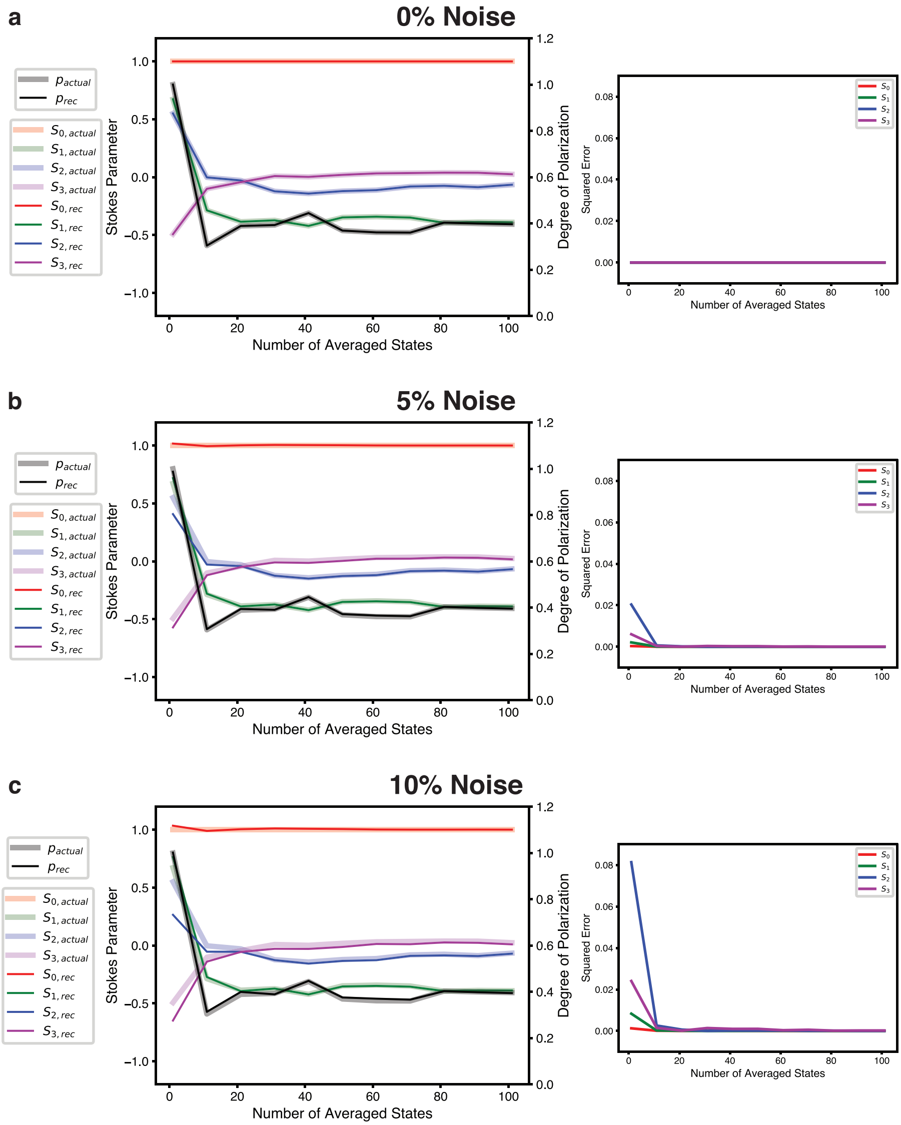

Supplementary Fig. 10: Stokes polarimetry reconstruction in simulation using the device from the main text under random mixed polarization inputs at a wavelength of $4.5\mu m$. **(a)** Reconstructed state locations broken down by Stokes parameter as well as degree of polarization compared to actual ones shown on the left with the associated squared error per Stokes parameter shown on the right. **(b)** Same plots as (a) but with 5% added noise. **(c)** Same plots as (a) but with 10% added noise.

10.11 Angular momentum device under different spin excitation

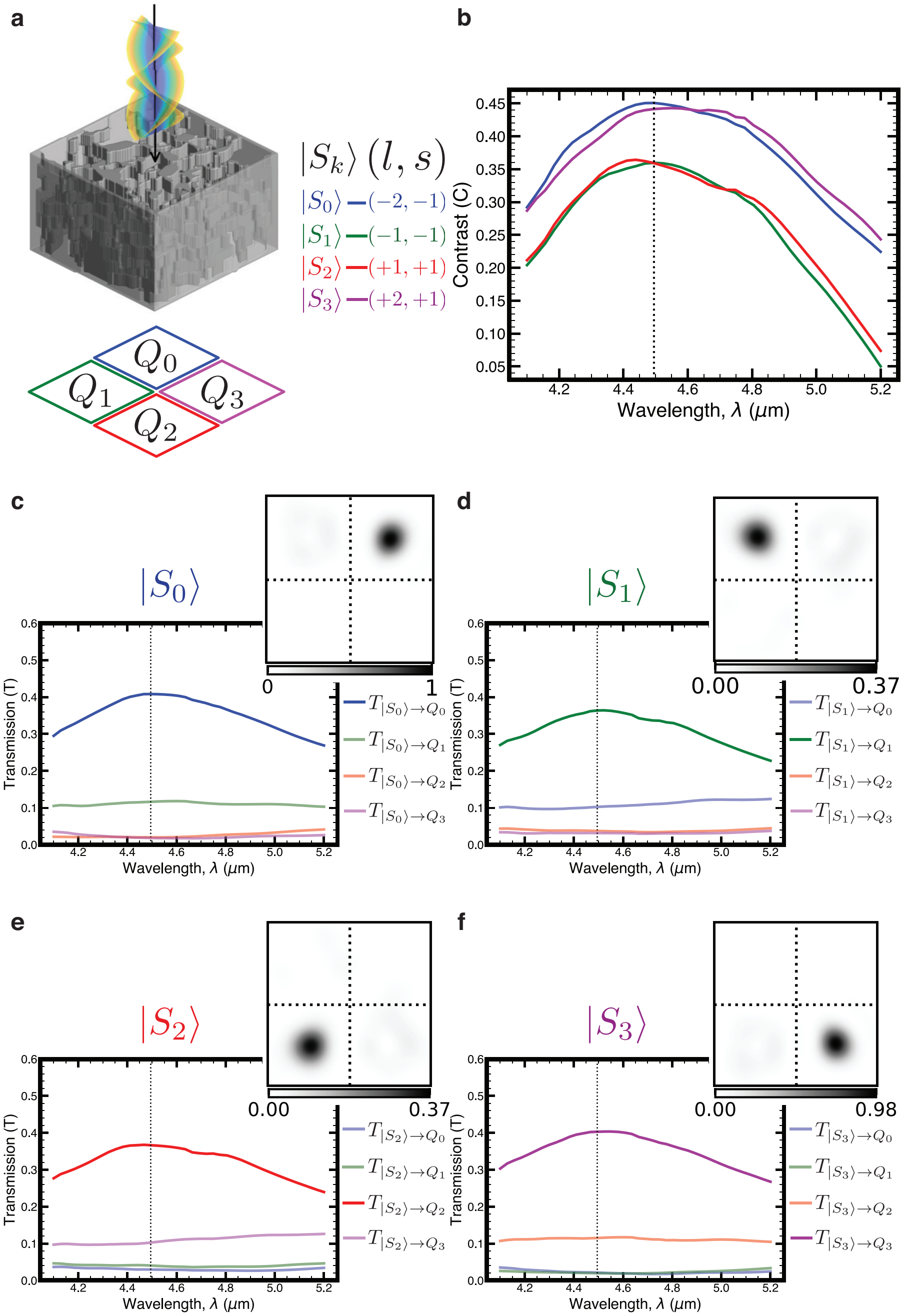
a
b
c
d
e
f
|S_k⟩ (l, s)
|S_0⟩ (−2, −1)
|S_1⟩ (−1, −1)
|S_2⟩ (+1, +1)
|S_3⟩ (+2, +1)
Q_0
Q_1
Q_2
Q_3
Contrast (C)
Wavelength, λ (μm)
Transmission (T)
|S_0⟩
|S_1⟩
|S_2⟩
|S_3⟩
0
1
0.00
0.37
0.00
0.37
0.00
0.98
T_{|S_0⟩→Q_0}
T_{|S_0⟩→Q_1}
T_{|S_0⟩→Q_2}
T_{|S_0⟩→Q_3}
T_{|S_1⟩→Q_0}
T_{|S_1⟩→Q_1}
T_{|S_1⟩→Q_2}
T_{|S_1⟩→Q_3}
T_{|S_2⟩→Q_0}
T_{|S_2⟩→Q_1}
T_{|S_2⟩→Q_2}
T_{|S_2⟩→Q_3}
T_{|S_3⟩→Q_0}
T_{|S_3⟩→Q_1}
T_{|S_3⟩→Q_2}
T_{|S_3⟩→Q_3}

Supplementary Fig. 11: Simulation of angular momentum sorting device for the same OAM, but different spin values than the design states. **(a)** Schematic of device and focal plane quadrants. **(b)** Contrast for sorting each state ($C \in [-1, 1]$) defined by the transmission of a state into the desired quadrant versus the transmission into the rest of the focal plane. For source k, $C_k = \frac{T_{|S_k\rangle \to Q_k} - \sum_{i \neq k} T_{|S_k\rangle \to Q_i}}{T_{|S_k\rangle \to Q_k} + \sum_{i \neq k} T_{|S_k\rangle \to Q_i}}$. **(c)** Transmission spectrum for ($l = -2$, $s = -1$) input with desired quadrant transmission in blue. Transmission is normalized by power through the device aperture with no device present. Inset: Intensity at focal plane (arbitrary units, but comparable to other intensity plots in figure). **(d-f)** Same plots as (c), but for ($l = -1$, $s = -1$), ($l = +1$, $s = +1$), ($l = +2$, $s = +1$), respectively.

10.12 Angular momentum device under different OAM excitation

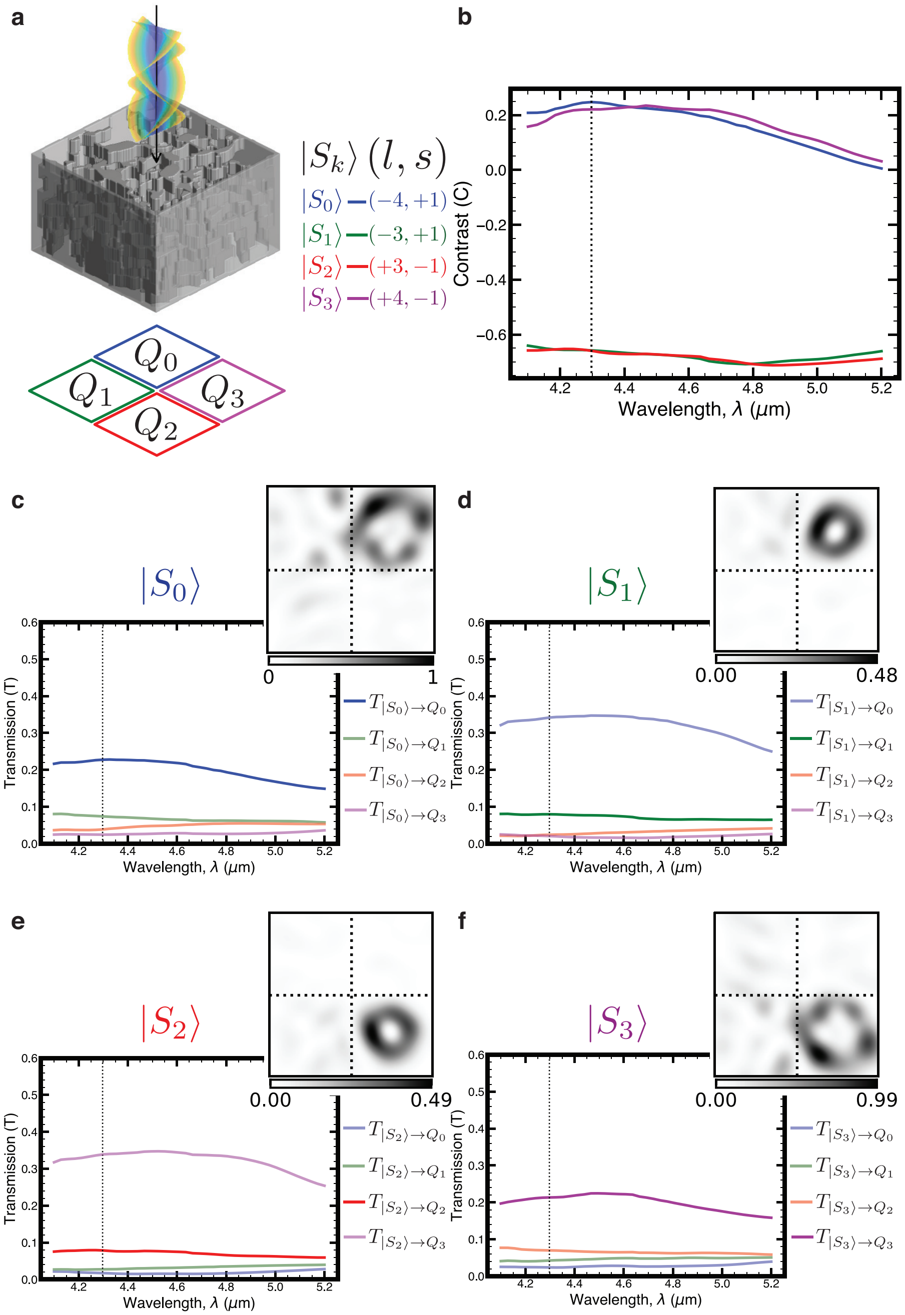
a
b
c
d
e
f
|S_k⟩ (l, s)
|S_0⟩ (−4, +1)
|S_1⟩ (−3, +1)
|S_2⟩ (+3, −1)
|S_3⟩ (+4, −1)
Q_0
Q_1
Q_2
Q_3
Contrast (C)
Wavelength, λ (μm)
Transmission (T)
|S_0⟩
|S_1⟩
|S_2⟩
|S_3⟩
0
1
0.00
0.48
0.49
0.99
T_{|S_0⟩→Q_0}
T_{|S_0⟩→Q_1}
T_{|S_0⟩→Q_2}
T_{|S_0⟩→Q_3}
T_{|S_1⟩→Q_0}
T_{|S_1⟩→Q_1}
T_{|S_1⟩→Q_2}
T_{|S_1⟩→Q_3}
T_{|S_2⟩→Q_0}
T_{|S_2⟩→Q_1}
T_{|S_2⟩→Q_2}
T_{|S_2⟩→Q_3}
T_{|S_3⟩→Q_0}
T_{|S_3⟩→Q_1}
T_{|S_3⟩→Q_2}
T_{|S_3⟩→Q_3}

Supplementary Fig. 12: Simulation of angular momentum sorting device for the same spin values, but different OAM than the design points. **(a)** Schematic of device and focal plane quadrants. **(b)** Contrast for sorting each state ($C \in [-1, 1]$) defined by the transmission of a state into the desired quadrant versus the transmission into the rest of the focal plane. For source k, $C_k = \frac{T_{|S_k\rangle \to Q_k} - \sum_{i \neq k} T_{|S_k\rangle \to Q_i}}{T_{|S_k\rangle \to Q_k} + \sum_{i \neq k} T_{|S_k\rangle \to Q_i}}$. **(c)** Transmission spectrum for ($l = -4$, $s = +1$) input with desired quadrant transmission in blue. Transmission is normalized by power through the device aperture with no device present. Inset: Intensity at focal plane (arbitrary units, but comparable to other intensity plots in figure). **(d-f)** Same plots as (c), but for ($l = -3$, $s = +1$), ($l = +3$, $s = -1$), ($l = +4$, $s = -1$), respectively.

References

[1] X. Zhou, Y. Hou, J. Lin, A review on the processing accuracy of two-photon polymerization. Aip Advances **5**(3), 030,701 (2015)

[2] L. Allen, M.W. Beijersbergen, R.J.C. Spreeuw, J.P. Woerdman, Orbital angular momentum of light and the transformation of Laguerre-Gaussian laser modes. Physical review A **45**(11), 8185 (1992)
