# Supplementary material for: 3D-patterned inverse-designed mid-infrared metaoptics
Source: Nat Commun. 2023 May 13;14:2768. doi: 10.1038/s41467-023-38258-2 (PMC10183040; doi:10.1038/s41467-023-38258-2)
Supplement: Supplementary file 2 — Peer Review File [file 41467_2023_38258_MOESM2_ESM.pdf]

nature portfolio

Peer Review File

3D-Patterned Inverse-Designed Mid-Infrared Metaoptics

Open Access This file is licensed under a Creative Commons Attribution 4.0 International License, which permits use, sharing, adaptation, distribution and reproduction in any medium or format, as long as you give appropriate credit to the original author(s) and the source, provide a link to the Creative Commons license, and indicate if changes were made. In the cases where the authors are anonymous, such as is the case for the reports of anonymous peer reviewers, author attribution should be to 'Anonymous Referee' followed by a clear attribution to the source work. The images or other third party material in this file are included in the article's Creative Commons license, unless indicated otherwise in a credit line to the material. If material is not included in the article's Creative Commons license and your intended use is not permitted by statutory regulation or exceeds the permitted use, you will need to obtain permission directly from the copyright holder. To view a copy of this license, visit http://creativecommons.org/licenses/by/4.0/.

Editorial Note: This manuscript has been previously reviewed at another journal that is not operating a transparent peer review scheme. This document only contains reviewer comments and rebuttal letters for versions considered at *Nature Communications*.

REVIEWERS' COMMENTS

Reviewer #1 (Remarks to the Author):

I have previously reviewed this manuscript. At that time, I found the work suitable for publication in *Nature Communications*. Since then, the authors have added some additional details to the manuscript, mainly in the supplement. These enhance the manuscript further, especially the discussion of the response of the OAM-sorting device to beams with OAM values lying outside of the set of four for which it was designed.

In short, I recommend this manuscript for publication in *Nature Communications*. I commend the authors on their very skilled design and fabrication work.

Reviewer #2 (Remarks to the Author):

I would be happy to recommend this work for publication in *Nature Communications* in the current form
